# The identification and functional annotation of RNA structures conserved in vertebrates

**DOI:** 10.1101/gr.208652.116

**Published:** 2017-08

**Authors:** Stefan E. Seemann, Aashiq H. Mirza, Claus Hansen, Claus H. Bang-Berthelsen, Christian Garde, Mikkel Christensen-Dalsgaard, Elfar Torarinsson, Zizhen Yao, Christopher T. Workman, Flemming Pociot, Henrik Nielsen, Niels Tommerup, Walter L. Ruzzo, Jan Gorodkin

**Affiliations:** 1 Center for non-coding RNA in Technology and Health (RTH), University of Copenhagen, DK-1870 Frederiksberg, Denmark;; 2 Department of Veterinary and Animal Sciences, Faculty of Health and Medical Sciences, University of Copenhagen, DK-1870 Frederiksberg, Denmark;; 3 Copenhagen Diabetes Research Center (CPH-DIRECT), Herlev University Hospital, DK-2730 Herlev, Denmark;; 4 Department of Cellular and Molecular Medicine (ICMM), Faculty of Health and Medical Sciences, University of Copenhagen, DK-2200 Copenhagen, Denmark;; 5 Department of Obesity Biology and Department of Molecular Genetics, Novo Nordisk A/S, DK-2880 Bagsværd, Denmark;; 6 Department of Biotechnology and Biomedicine, Technical University of Denmark, DK-2800 Kongens Lyngby, Denmark;; 7 Allen Institute for Brain Science, Seattle, Washington 98109, USA;; 8 School of Computer Science and Engineering and Department of Genome Sciences, University of Washington, Seattle, Washington 98195, USA;; 9 Fred Hutchinson Cancer Research Center, Seattle, Washington 98109, USA

## Abstract

Structured elements of RNA molecules are essential in, e.g., RNA stabilization, localization, and protein interaction, and their conservation across species suggests a common functional role. We computationally screened vertebrate genomes for conserved RNA structures (CRSs), leveraging structure-based, rather than sequence-based, alignments. After careful correction for sequence identity and GC content, we predict ∼516,000 human genomic regions containing CRSs. We find that a substantial fraction of human–mouse CRS regions (1) colocalize consistently with binding sites of the same RNA binding proteins (RBPs) or (2) are transcribed in corresponding tissues. Additionally, a CaptureSeq experiment revealed expression of many of our CRS regions in human fetal brain, including 662 novel ones. For selected human and mouse candidate pairs, qRT-PCR and in vitro RNA structure probing supported both shared expression and shared structure despite low abundance and low sequence identity. About 30,000 CRS regions are located near coding or long noncoding RNA genes or within enhancers. Structured (CRS overlapping) enhancer RNAs and extended 3′ ends have significantly increased expression levels over their nonstructured counterparts. Our findings of transcribed uncharacterized regulatory regions that contain CRSs support their RNA-mediated functionality.

Computational analyses have suggested many conserved structured RNAs in vertebrate genomes (Washietl et al. 2005; Torarinsson et al. 2008; Parker et al. 2011; Smith et al. 2013). Recent transcriptome-wide experiments also support a diverse RNA structure landscape (Ding et al. 2014; Rouskin et al. 2014; Wan et al. 2014; Aw et al. 2016; Lu et al. 2016; Sharma et al. 2016). These experiments, however, do not broadly exploit the phylogenetic context in which functionally important RNAs appear, especially compensatory base-pair changes. Furthermore, previous computational screens for conserved RNA structures (CRSs) have focused on sequence-based alignments (Gorodkin et al. 2010), although structural alignments more sensitively capture evolutionarily CRSs (Wang et al. 2007). While structure is known to be critical to the biogenesis or function of many noncoding RNAs (ncRNAs), it remains unclear how ubiquitous a role conserved structures play. For example, a recent experiment mapping RNA duplexes in living human and mouse cells (Lu et al. 2016) reported conserved structured RNA domains in several long ncRNAs (lncRNAs), including *XIST* and *MALAT1*, while in silico studies based on single sequence folding and sequence-based alignments (Managadze et al. 2011) have indicated that RNA secondary structures are depleted in lncRNAs (Ulitsky and Bartel 2013). The low sequence conservation of most lncRNAs, while complicating identification of CRSs, does not preclude their existence, such as in telomerase RNA (structurally similar across vertebrates despite human–mouse sequence identity [SI] of ∼60%).

Given these difficulties in detecting conserved structures, the accuracy of computational screening methods is a prime concern. Here, we present a carefully designed discovery pipeline and a significantly improved scoring scheme, with careful control for technical factors such as dinucleotide composition and GC content, with the goal of reducing the false-discovery rate (FDR) of the predicted CRSs.

Regulatory features of RNA structures have been extensively studied in bacteria (Waters and Storz 2009), but the vast landscape of RNA regulatory elements in vertebrates remains largely uncharacterized. Conservation, structural or otherwise, typically implies function but does not tell us *what* function. RNA structures are known to be involved in gene regulation through transcript stabilization (Goodarzi et al. 2012), interaction with RNA binding proteins (RBPs) (Ray et al. 2013), and other processes. While most RBPs contain a few RNA binding domains, the contextual features that regulate RBP binding are often of limited sequence specificity and are not well known. Some RBPs specifically bind double-stranded RNAs (dsRBPs); examples include DGCR8 and DICER1, important in siRNA and microRNA biogenesis (Macias et al. 2012; Rybak-Wolf et al. 2014), and STAU1 in regulated RNA decay (Kim et al. 2005). Other RBPs bind unpaired nucleotides exposed in loops and additional secondary structure elements (Lunde et al. 2007).

The complexity of the vertebrate transcriptome had been underestimated for decades, but the advent of high-throughput sequencing has enabled the identification of many new transcripts. For example, expression upstream of promoters (Seila et al. 2008; Preker et al. 2011) and RNAs transcribed from enhancers (eRNAs) (Andersson et al. 2014a; Arner et al. 2015) have recently been recognized to be common, but are often viewed as transcriptional by-products at accessible genomic sites, especially because of generally rapid degradation by the nuclear exosome (Almada et al. 2013; Jensen et al. 2013; Ntini et al. 2013). However, experimental data increasingly suggest functional roles for these transcripts (Di Giammartino et al. 2011; Rinn and Chang 2012; Li et al. 2013). As another example, the majority of human genes are alternatively cleaved and polyadenylated, and these alternative isoforms differ in their stability, localization, and translational efficiency (Elkon et al. 2013). Many regulatory elements are located in untranslated regions of mRNAs (UTRs) and recognized by RBPs (Berkovits and Mayr 2015). Many are structured, as indicated by the large repertoire of RNA structure families in Rfam (Nawrocki et al. 2015). The structures themselves might even affect alternative polyadenylation and stability (Di Giammartino et al. 2011) or coexist in downstream independent transcripts, because RNA polymerase II does not cease transcription at the poly(A) site (Glover-Cutter et al. 2008).

In short, many noncoding genomic regions, including gene regulatory regions, are transcribed and may host functionally important structures, but their superficial lack of sequence conservation might systematically bias against discovery of RNA structures, thus motivating our main aim: genome-wide exploration of CRSs based on structure-aware multispecies alignments. To assess our in silico CRS predictions, we present extensive correlations with public and novel experimental data in two directions. The first assesses the accuracy of our computational predictions. For example, we used RNA structure probing experiments to test our predictions of structure conservation between human and mouse and used RNA-seq and RT-qPCR to test human–mouse coexpression. A second direction assesses potential CRS roles in specific functions, for example, RBP interactions and enhancer activity.

## Results

### Identification of CRSs by local structural alignments

To identify CRSs, we extracted sequences from MULTIZ alignments (MAs) from 17 vertebrates (hg18) (Blanchette et al. 2004), collectively corresponding to ∼50% of the human genome (Methods). RNA structure predictions were made in these putatively orthologous sequence sets by CMfinder, which locally and structurally aligns a set of unaligned sequences, discarding apparently irrelevant ones (Yao et al. 2006). CMfinder is not constrained by the initial sequence-based alignment or by predefined window sizes. It has been broadly successful, e.g., aiding in the discovery of large bacterial ncRNAs (Weinberg et al. 2009) and of numerous ribozymes and riboswitches (Weinberg et al. 2007). Predictions from the 17-species analysis were extended to the 100-species tree (hg38) (Methods).

**Figure 1.**
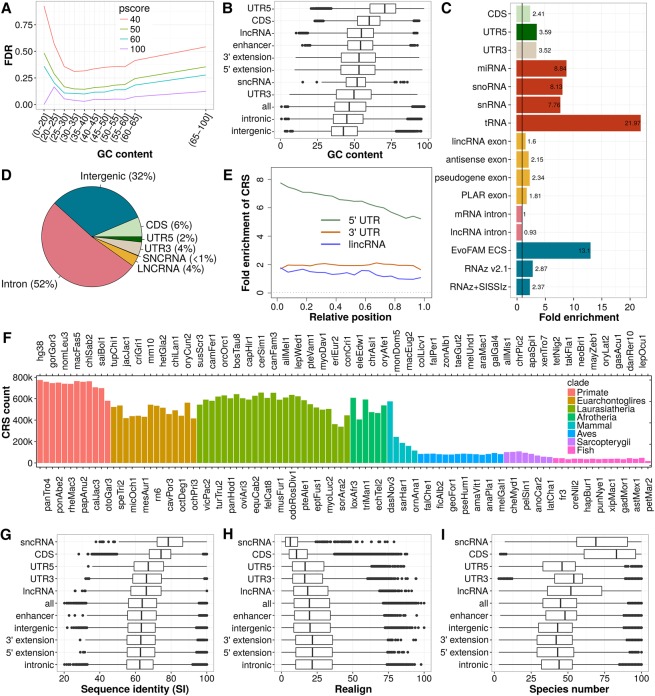
Performance assessment, genomic distribution, and conservation of CRS predictions. (*A*) Mean FDR of CRSs for different CMfinder score (pscore) cutoffs and GC-content intervals. FDR calculation is based on SISSIz (Gesell and Washietl 2008) simulated alignments. The large decrease in FDR observed between pscore cutoff 40 and 50 motivated us to base all further analyses on pscore ≥ 50. The mean FDR covering all ranges of GC content is 15.8. (*B*) GC content of CRS region alignments. (*C*) Fold enrichment of CRS regions for biotypes and previous computational RNA structure screens in vertebrates (blue). (*D*) Absolute CRS region coverage of biotypes. (*E*) Relative position of CRS regions over noncoding biotypes presented as fold enrichment of CRS regions in bins, each 5% (considering only exons) of the feature's (UTR or gene) length. The trend of decreasing number of structures from 5′ to 3′ is common to 5′ UTRs and lncRNAs. (*F*) Number of CRSs conserved in the 100-species tree. (*G*) Average pairwise sequence identity (SI) of CRS region alignments over the 17 representative genomes in the phylogenetic tree. (*H*) Realignment (calculated as in Torarinsson et al. 2008) compares the 17-species MULTIZ alignment blocks (hg18) to corresponding structure-based alignments of CRS regions (17-way subalignments extracted from our 100-species/hg38 results, as described in Methods). (*I*) Species number of CRS region alignments. In *B*,*G*, and *I*, the CRSs of highest GC content, SI and species number, respectively, are used as representatives of a CRS region, and in *H* the CRSs of lowest realignment are used as representatives. The biotypes in *G*, *H*, and *I* are sorted by their median SI.

We predicted 773,850 CRSs (pscore ≥ 50; Methods) covering 515,506 CRS regions (genomic regions of overlapping CRSs). We estimated our CRS FDR to be 14.1 ± 5.1% within the most common GC-content range of 20%–65% (using a dinucleotide controlled and GC-content-corrected phylogenetic null model) (Supplemental Methods), while the top 20% of CRSs ranked by pscore have an estimated FDR < 10% (Fig. 1A; Supplemental Fig. S1). A GC-content-specific FDR is important because GC contents vary strongly among CRSs and across different biotypes (genomic locations of similar characteristics) (Fig. 1B).

Seventy-two percent of CMfinder-predicted base pairs agree with an independent in vivo biological assessment by genome-wide structure probing (Rouskin et al. 2014) (Methods). The even higher agreement between our in silico predictions and their in vitro data (Supplemental Fig. S2) further supports our methodology; neither in silico nor in vitro considers the cellular environment, including protein binding.

Predicted CRSs average 71 ± 46 bp in length and cover 36.5 million bases (∼2.6%) of the human input sequence. On average, they are conserved in 45 ± 19 species of the 100-species tree (Fig. 1F,I) with deeper conservation in sncRNAs and mRNAs (coding sequences [CDS]). CRSs regions are mostly intronic or intergenic (Fig. 1D; annotation sources are listed in Supplemental Material) and are enriched for small ncRNAs (sncRNAs; including 230 precursor-microRNAs and 199 snoRNAs) and UTRs (Fig. 1C; Supplemental Table S1). They overlap 36% of the 1067 structured (base pair content >30%) Rfam (Burge et al. 2013) input sequences, comprising mostly sncRNAs and *cis*-regulatory structures in UTRs. The majority of lncRNAs lack CRSs (Fig. 1C; Supplemental Fig. S3), consistent with previous observations (Ulitsky and Bartel 2013). Nonetheless, in addition to known examples such as tRNA-like structures in *MALAT1* and *NEAT1* (Supplemental Fig. S4; Zhang et al. 2014), many lncRNAs host CRSs, including 22% of screened lncRNAs annotated in GENCODE v25 (Harrow et al. 2012), 19% of lncRNAs annotated from RNA-seq data (PLAR) (Hezroni et al. 2015), 31% of anti-sense ncRNAs, and 30% of processed pseudogenes. Within lncRNAs, CRS density decreases from 5′ to 3′ (Fig. 1E). A small number of CRSs (13,535) outside annotated coding sequences (GENCODE) hold coding potential according to PhyloCSF (Lin et al. 2011). Although 167,000 CRSs (21.6%) overlap repeats flagged by RepeatMasker v4.0.5 or TandemRepeatFinder v4.0.4 (Smit et al. 2013), almost all repeat families are depleted of CRSs (Supplemental Table S2). SINE, LINE, and simple repeats comprise the majority of CRS/repeat overlaps, including 1572 CRSs that overlap *Alu* elements.

Evolutionary constraints on RNA structure do not necessarily coincide with evolutionary constraints on sequence. In particular, ∼50% of CRSs fall outside of conserved elements identified by phastCons (Siepel et al. 2005) in the 100-species alignment, and CRS regions of low SI showed a higher degree of realignment when structure was taken into account (Fig. 1G,H). CRS regions within annotated coding sequences, UTRs, or targets of RNA- and DNA-binding proteins (e.g., transcription factor [TF] binding sites) generally show higher SI and less realignment. In contrast, intronic CRS regions and ones within 2 kb upstream (“5′ extension”) or within 2 kb downstream (“3′ extension”) to mRNAs and lncRNAs showed lower SI and significantly more realignment (*P* < 10^−6^, *t*-test) (Fig. 1G,H). Expression extending beyond annotated UTRs and lncRNAs was repeatedly observed in transcriptomic data, which is addressed below. Lower SI in gene extensions (∼64% SI) may indicate faster adaptation of these RNA structures to novel functions than those in either lncRNAs (∼66% SI) or mRNAs (CDSs and UTRs ∼70% SI).

### Purifying selection

Despite their somewhat low SI, CRSs show signatures of purifying selection. First, nucleotide distances between primates and rodents are lower in CRSs than in nearby ancestral repeats (ARs) or intergenic loci (*P* < 10^−12^, two-sided Kolmogorov-Smirnov test) (Supplemental Fig. S5A,B). Nucleotide substitution patterns for ∼62% of the 26,000 CRSs with orthologs and appropriate nearby control sequences are improbable under neutralist models (Methods). Second, CRSs are enriched within DNA that has been subject to purifying selection with respect to indels (Lunter et al. 2006) (*P* < 10^−10^, one-sided *Z*-test, Benjamini-Hochberg [BH] corrected; Supplemental Fig. S5D). Third, the minor allele frequencies (MAFs) in a large human population (The 1000 Genomes Project Consortium 2015) of 271,000 CRSs ≥ 2 kb away from known mRNAs are significantly lower than in regions 5 to 10 kb up- and downstream (*P* < 10^−16^, Mann–Whitney *U* test) (Supplemental Methods).

### Colocalization between CRSs and conserved RBP binding sites

**Figure 2.**
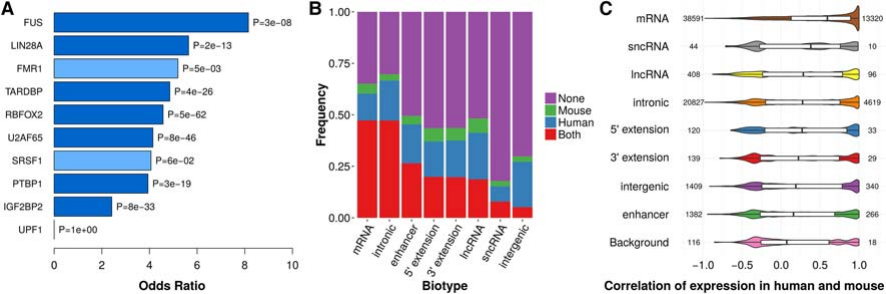
Human and mouse conservation of CRS regions is reflected by binding sites of RBPs and expression. (*A*) Seven of 10 RBPs display enrichment of CRSs in conserved binding sites (*P* < 10^−7^, FET). Significant enrichments are colored dark blue; light blue were not significant. (*B*) A relatively large number of CRSs (146,670) are expressed in both human and mouse (red bars) over four tissues (heart, liver, diencephalon/forebrain, and cerebellum/hindbrain) with comparable total RNA-seq data (Methods). In total, 157,136 CRSs are expressed in both human and mouse in total RNA-seq or poly(A) RNA-seq (Supplemental Fig. S7). CRSs with an empirical *P*-value < 0.01 were assigned an “expressed” state. We considered only 433,327 of 543,390 human–mouse conserved CRSs that have the same biotype in both species. Note that “5′ extension” and “3′ extension” refer to 2-kb regions upstream of and downstream from UTRs and lncRNAs; UTRs themselves are included in “mRNA.” (*C*) Expression correlation between human and mouse for different biotypes was measured by Pearson's correlation coefficient *r* of expression levels in poly(A) RNA-seq (six tissues: testis, liver, kidney, heart, cerebellum, and brain). “Background” is sampled over the input MA blocks with human–mouse conservation not overlapping the other biotypes. The number on the *left* of violin plots is total number of measured CRSs with expression in at least two tissues, and the number on the *right* side is number of CRSs with *r* > 0.8.

RNA targets from in vivo CLIP experiments for 67 human RBPs overlap 102,000 CRS regions (Methods), which correlates with CRS enrichment for binding sites of 76% of human RBPs after stratifying for GC content and SI (*P* < 10^−10^, one-sided *Z*-test, BH-corrected). For most RBPs, CRSs were enriched around binding sites in regions having GC content >40% or high SI (Supplemental Fig. S6). For some RBPs, CRSs were also enriched in low SI regions. Examples include the IGF2 mRNA binding protein family (IGF2BPs), whose three mammalian paralogs contain two RRM and four KH domains, all putatively involved in RNA binding with limited sequence specificity. Human–mouse conserved CRSs associated to IGF2BP2 binding in human also showed RNA binding in mouse, supporting their functional roles in binding site recognition. More generally, seven of 10 studied RBP orthologs in mouse (Methods), including IGF2BP2, are enriched for binding sites overlapped by CRSs both in human and mouse (*P* < 10^−7^, Fisher's exact test [FET]) (Fig. 2A). Alternative splicing is an example of RBP recruitment modulated by the kinetics and thermodynamics of RNA structure (Raker et al. 2009), and in our study, the binding sites for the splicing factors RBFOX2, HNRNPA1, and PTBP1 were enriched for CRSs near splice sites (*P* < 0.001, FET).

### Conserved expression in human and mouse

Cross-species conserved transcriptional activity of CRSs can imply conserved biological function. We selected closely matched human/mouse RNA-seq samples from 10 tissues (Supplemental Methods; Supplemental Table S8). In both species, the highest expression levels of CRSs occurred within exons of mRNAs and lncRNAs (Supplemental Fig. S7). Using an empirical *P*-value calculated from background expression (Methods), conserved transcriptional activity (*P* < 0.01) was supported for ∼36% of the shared human–mouse CRSs having concordant biotypes in both species (Fig. 2B). This was dominated by CRSs of mRNAs and introns (∼50%), but CRSs within enhancers, 5′ extensions, 3′ extensions, and lncRNAs were also well represented (>20% of shared CRSs of each biotype were coexpressed). This overlap in expression remains evident in individual tissues; e.g., among CRSs expressed in at least two tissues in both species, 23% showed strongly correlated expression (Pearson's correlation *r* ≥ 0.8), including 164 from lncRNAs, 788 from enhancers, and 780 intergenic CRSs (Methods) (Fig. 2C; Supplemental Fig. S7G). Despite relatively small numbers, CRSs within mRNAs, lncRNAs, sncRNAs, and 5′ and 3′ extensions have significantly larger cross-species coexpression than background (*P* < 0.05, one-sided Mann–Whitney *U* test, BH-corrected). For example, the lncRNA *MIR22HG*, hosting several CRS regions in addition to the microRNA *MIR22*, is expressed in all tissues considered here, and the noncoding testis development related 1 (*TDRG1*), exclusively expressed in testis of both human and mouse, has a large CRS region of low SI (∼60%) extending beyond its annotated 3′ end.

### A CaptureSeq experiment detects weakly expressed structured RNAs

**Figure 3.**
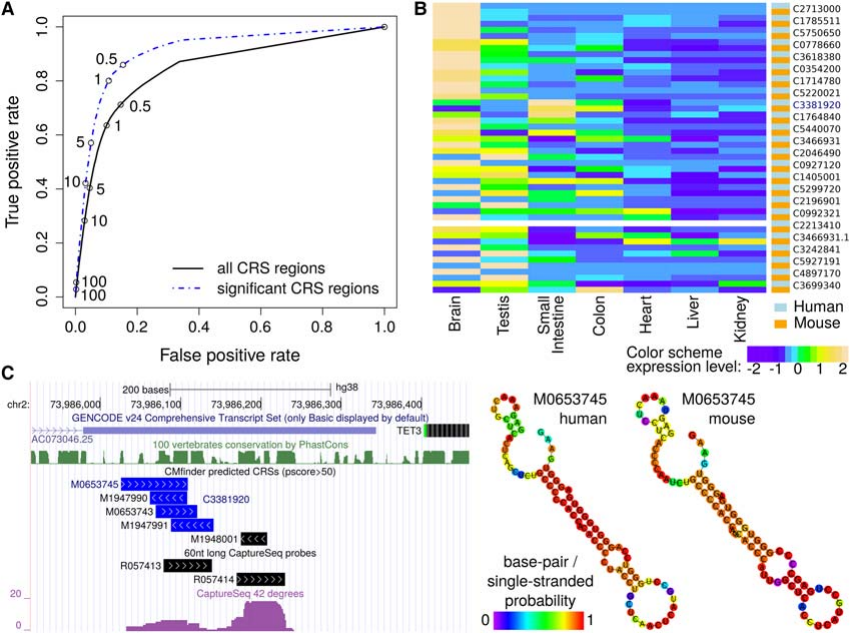
CaptureSeq and qRT-PCR show conserved expression of CRSs. (*A*) ROC curve of CRS region detection in brain based on public poly(A) RNA-seq defined by different CPM/RLE cutoffs (numbers on the curves) using the CRS region detection through CaptureSeq in fetal brain as the gold standard. (*B*) Expression profiles of 23 CRS regions were measured with qRT-PCR (normalized by CRS regions) in seven tissues in both human and mouse. The CRS regions have weakly conserved primary sequences and were expressed in the CaptureSeq (*P* < 0.1). The CRS regions are sorted by decreasing Pearson's correlation coefficients of expression profiles between human and mouse. (*C*) The CRS region C3381920 is located in the 3′ end of the lncRNA *AC07304.25*. Despite no expression in brain in publicly available total and poly(A) RNA-seq data, it showed up in human brain in both CaptureSeq and qRT-PCR. Common expression in human and mouse was observed in the gastrointestinal tract (small intestine and colon; see *B*). Region C3381920 contains the CRS M0653745 whose structure is highly conserved between human and mouse. Color code in human and mouse structures is base-pair probabilities calculated by the Vienna RNA package (Lorenz et al. 2011).

Almost 50% of the CRS regions overlap with abundant transcription in publicly available total RNA-seq and poly(A) RNA-seq from 16 and 19 human tissues, respectively (empirical *P*-value < 0.01 and normalized count per million reads (CPM/RLE) >1 in ≥2 tissues; Methods). In standard RNA-seq experiments, however, weakly expressed transcripts are undetectable or indistinguishable from nonspecific transcription (Lin et al. 2014). To improve detection sensitivity, we designed capture probes for 77,320 CRS regions (Methods, Supplemental Methods) and coupled the capture with an RNA-seq experiment (CaptureSeq). In Figure 3A (and Supplemental Fig. S8), we observe good agreement between the expression of the captured CRS regions and publicly available data. In human fetal brain alone, 8385 CRS regions were significantly expressed (*P* < 0.1; Methods, Supplemental Methods), including 662 transcripts that were previously not detected in brain (less than one CPM/RLE; Methods). The majority of these CRS regions are located in UTRs or UTR extensions, another 115 CRS regions fall in intergenic regions, 205 in lncRNAs and 50 in sncRNAs. Many of these regions (1475) are weakly conserved in sequence (SI < 60%). Examples include human–mouse CRSs that overlap the known conserved stem loop in exon 4 of *XIST* (Fang et al. 2015), the highly structured telomerase RNA element, the brain-specific *MIR9-2* host gene *LINC00461*, and the *SH3RF3* anti-sense RNA 1 (*SH3RF3-AS1*).

### qRT-PCR shows correlated expression profiles in human and mouse

To further explore conserved expression between human and mouse presented above, we compared the expression of selected CRSs across seven tissues in both species via qRT-PCR. We studied 23 CRS regions of low to medium SI and with weak expression in publicly available brain samples (Methods) covering five novel transcripts, five transcripts close to annotated mRNAs, eight recently annotated extensions of UTRs, and five lncRNAs (Supplemental Table S3). These regions were detected in at least 80% of the examined tissues despite low expression levels, and nine CRS regions showed strong coexpression between human and mouse (Pearson's correlation *r* > 0.8) (Fig. 3B). For example, Figure 3C shows a structure conserved in 59 species with SI of 75% that was predicted in the tissue-specific lncRNA *AC073046.25*. Thus, our CaptureSeq strategy identified (and qRT-PCR partially validated) conserved expression profiles of CRSs with low abundance and low sequence conservation, leading to putative functional genes.

### Structure probing shows CRSs in human and mouse

**Figure 4.**
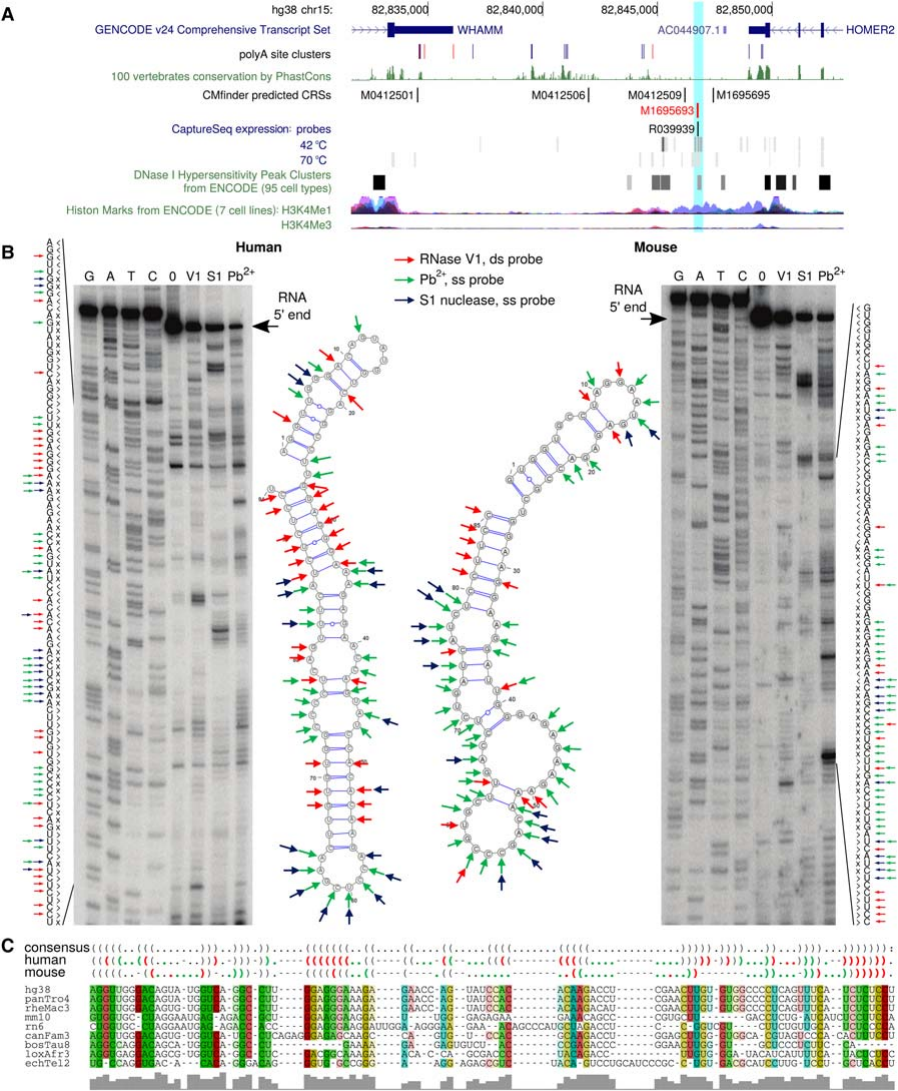
In vitro RNA structure probing in human and mouse shows conserved structure of CRS M1695693. FDR is 11.0% and SI of the nine-species (filtered from the 17-species tree) structural alignment is 48% (45% between human and mouse). The CRS is located between the 3′ UTRs of *HOMER2* (minus strand) and *WHAMM* (plus strand; Chr 15: 8284671–82846804). It overlaps a DNase hypersensitive site (DHS) (ENCODE) and has the typical chromatin signatures of enhancers, namely, enrichment of H3K4me1 and reduced enrichment of H3K4me3, all indicators for a transcribed regulatory region. However, CAGE data from FANTOM5 did not support this hypothesis; instead, poly(A) site clusters (Gruber et al. 2016) suggest an extended 3′ UTR of *HOMER2*. (*A*) Genomic tracks. (*B*) Structure probing results in human and mouse, where red marks base-paired nucleotides (ds), and green and blue mark single-stranded nucleotides (ss). (*C*) CMfinder's structural alignment, predicted consensus RNA secondary structure, and predicted individual structures in human and mouse as dot-bracket notation. The probing results are overlapped with the in silico predictions by their color code.

To investigate whether CRSs of low SI were indeed structurally conserved, we performed RNA structure probing (Methods) of homologous human and mouse sequences of 10 CRSs, selected based on their low FDR (∼10%), low SI, and qRT-PCR–validated coexpression in human and mouse brain. In vitro transcription yielded four CRS pairs suitable for structure probing as determined by native gel electrophoresis (Supplemental Table S4). In all four cases, there was strong consistency between the predicted structures and the experimental analyses (Fig. 4; Supplemental Fig. S9). The CRSs originated from the 3′ end of the brain-specific mRNA *KCNG2*, the short 5′ UTR of brain-specific *EOMES*, lncRNA *MIR4697HG* (the hosted microRNA was not probed), and CRS candidate M1695693, found downstream from the annotated 3′ end of *HOMER2* (a postsynaptic density scaffolding protein). Poly(A) signals from RNA-seq (Gruber et al. 2016) supported an extended 3′ UTR of *HOMER2* covering the CRS region (Fig. 4A). Despite 45% SI between human and mouse, the probing showed that the two dissimilar sequences can fold into closely related structures largely in agreement with the structural alignment (Fig. 4B,C).

### RNA structures are enriched within gene regulatory regions

**Figure 5.**
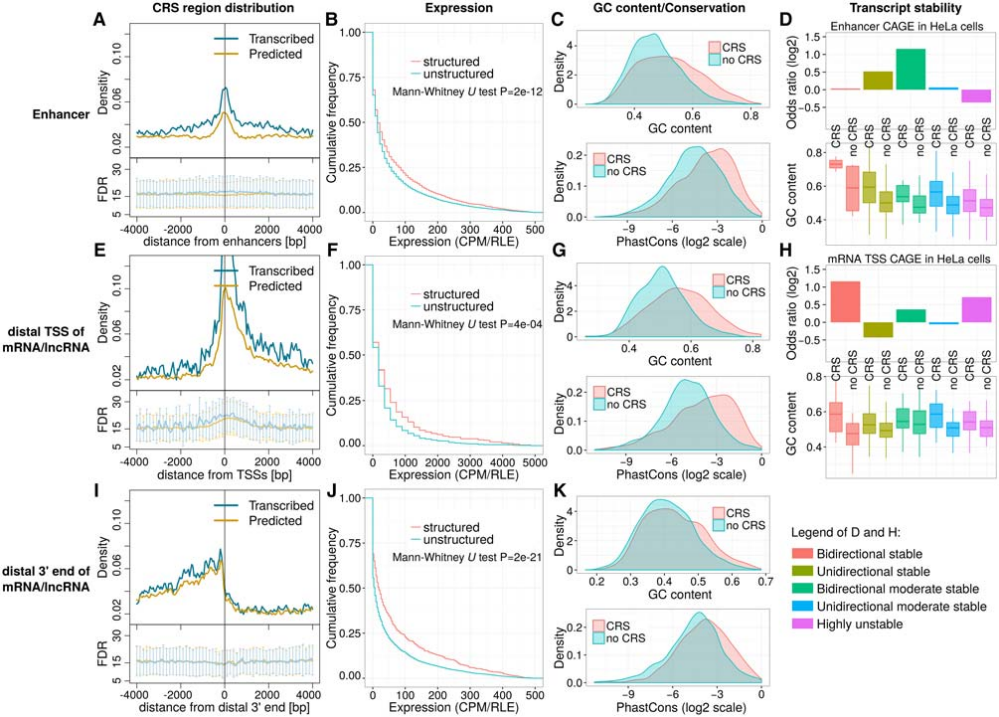
Coverage and expression of CRS regions in gene regulatory regions. The figure's three rows describe regions surrounding (1) enhancers (*A*–*D*), (2) most distal TSS of mRNAs/lncRNAs (*E*–*H*), and (3) most distal 3′ end of mRNAs/lncRNAs (*I*–*K*), respectively. (*A*,*E*,*I*) Plot density of CRS regions near those features: counts in 50-bp windows normalized by the number of features. “Predicted” curves (orange) reflect all CRS regions; “transcribed” curves (blue) reflect the subset supported by unannotated transcription boundaries. *Lower* subpanels show estimated FDRs (mean, SD) of those predictions. All other panels are based on the “transcribed” subset; for details, see Methods section “Definition of Gene Regulatory Regions” and Supplemental Figure S11. In summary, expression is based on the following: (*B*,*C*) CAGE TSS near enhancers, (*F*,*G*) CAGE TSS upstream anti-sense w.r.t. mRNA/lncRNA, and (*J*,*K*) active poly(A) sites downstream sense w.r.t. mRNA/lncRNA. “Structured”/“CRS” denote regions that overlap CRSs; “unstructured”/“no CRS” do not. *(B*,*C*,*F*,*G)* Total RNA-seq in fetal human cerebellum (technical replicate two of experiment ENCSR000AEW; ENCODE Phase 3). (*J*,*K)* Poly(A) RNA-seq of human brain (HBM). (*B*,*F*,*J*) Expression levels are in counts per million after cross-experiment relative log expression normalization (CPM/RLE). (*C*,*G*,*K*) GC content and phastCons (from 100-species MULTIZ alignments) of expressed structured (CRS) versus unstructured regions (no CRS). Expressed regions were defined by empirical *P*-value < 0.01 and CPM/RLE ≥ 1. (*D*,*H*) Transcript stability at ENCODE HeLa DHSs, as described in Andersson et al. (2014b), and GC content of structured (CRS) and unstructured regions (no CRS). Odds ratios quantify how strongly stability is associated with CRS overlap.

A substantial fraction (∼40%) of the 433,000 intergenic and intronic CRS regions are located at TF binding sites, DNase hypersensitive sites (DHSs), or loci exhibiting promoter- and enhancer-specific chromatin marks, all suggesting regulatory activities. This prompted a more detailed analysis of the approximately 30,000 CRS regions found within (1) 1 kb of enhancers, (2) 5′ extensions, or (3) 3′ extensions of mRNAs and lncRNAs, collectively called gene regulatory regions here. We attempted to control for several potential confounding factors in these regions; e.g., their comparatively rapid evolution (lower SI than other CRSs (*P* < 10^−7^, two-sided Mann–Whitney *U* test) (Fig. 1G) complicates the use of phylogenetic measures (Ponting 2008), and the significantly higher GC content compared to other CRSs (*P* < 10^−78^, two-sided Mann–Whitney *U* test) makes GC content correction especially important. Since approximately half of these CRSs colocalize with TF binding sites, we considered, but ruled out, the possibility that palindromic DNA sequences of TF binding sites might give rise to spurious CRS predictions (Supplemental Fig. S10). Our study nonetheless reveals not only structure conservation in these gene regulatory regions but also shows CRSs to be sharply enriched (Fig. 5A,E,I; Supplemental Table S5), even after allowing for a slightly higher GC-content-corrected FDR for predictions around TSSs (Fig. 5E, lower subpanel).

**Figure 6.**
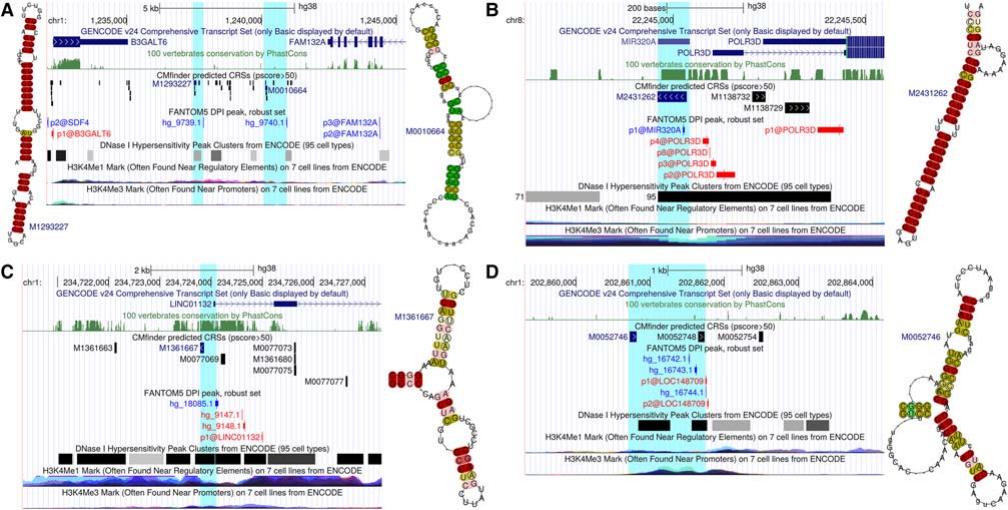
Example CRSs in gene regulatory regions are supported by unannotated transcript boundaries. (*A*) Two intergenic enhancers in a highly structured region of low SI (CRS M1293227 is only conserved in primates) between two gene 3′ ends. (*B*) *MIR320A* is upstream of *POLR3D* TSS. (*C*) Anti-sense transcription at the promoter of the lncRNA *LINC01132* has enhancer-like chromatin signatures. (*D*) Intergenic enhancer with unidirectional stable transcription from the minus strand as measured by control and exosome-depleted HeLa cells (Andersson et al. 2014b). Color code in consensus structures is the level of base-pair conservation in the structure-based alignments.

To further preclude false signal from DNA motifs unrelated to RNA structure, we analyzed in greater detail the subset of CRSs located near experimentally determined but unannotated transcript boundaries (TSSs and poly(A) sites). By using DHSs, CAGE measured TSSs, and characteristic chromatin marks (Methods) (Supplemental Fig. S11), we found 10,110 enhancer transcripts of which 2862 contained CRSs (with >50% of their length downstream from the TSS) (fold enrichment FE = 1.67, *Z*-test *P* < 10^−185^) (Fig. 5A). Figure 6A shows two putative eRNAs in a structured intergenic region of low SI between two protein-coding genes involved in glucose metabolism. We also found 1077 mRNAs and 129 lncRNAs with transcripts upstream of and anti-sense to their annotated TSS, of which 337 contained CRSs (FE = 1.83, *Z*-test *P* < 10^−40^) (Fig. 5E). However, upstream sense TSSs (≤1300 bp from annotated TSSs) (Supplemental Fig. S11) for a considerably larger number of mRNAs (2530) and lncRNAs (519) plausibly reflect alternative TSSs (unannotated in GENCODE), and 24% of these 5′ extensions contained CRSs. In four instances, the CRS region overlapped pre-miRNAs (*MIR320A*, *MIR34B*, *MIR219A1*, *MIR4665*), suggesting exploitation of TSS upstream transcripts (of either orientation) to coregulate multiple components of specific pathways. For example, *MIR320A* is transcribed anti-sense to and located in the promoter region of *POLR3D* and regulates TFs of *POLR3D* (Fig. 6B; Kim et al. 2008). Figure 6C shows a bidirectionally transcribed locus of unknown function at the promoter of a lncRNA. Active poly(A) site data (Gruber et al. 2016) revealed 2885 mRNAs and 1260 lncRNAs with an alternative poly(A) site between 50 bp and 2 kb downstream from the most distal GENCODE annotated 3′ end. Of these, 543 mRNAs and 203 lncRNAs had a predicted CRS within their 3′ extension, reflecting a modest FE of CRSs in the mRNA extensions (FE = 1.15, *Z*-test *P* = 0.0001) (Fig. 5I). An example is CRS M1695693, which is likely linked to *HOMER2* as discussed above and in Figure 4. In all three regulatory regions, we saw higher density of CRSs in loci supported by experimentally defined transcript boundaries (Fig. 5A,E,I, “transcribed” curve).

### CRSs impact transcription of enhancers and 3′ extensions

We then explored whether the observed enrichment of predicted CRSs in regulatory regions can be linked to transcription, focusing on regions with experimentally defined transcript boundaries. Note, however, that regulatory programs are highly tissue specific, emphasizing that our conservative candidate list may only represent a subset of the transcriptomic landscape. Specifically, we examined total RNA-seq and poly(A) RNA-seq of 19 and 16 tissues, respectively (Methods). For (1) transcription around enhancers, (2) TSS-upstream transcription, and (3) transcription of 3′ extensions, CRS-overlapping (“structured”) transcripts show significantly higher expression levels in all tested tissues compared unstructured ones (*P* < 0.001, one-sided Mann–Whitney *U* test, BH-corrected) (Fig. 5B,F,J). However, we also observed that GC content and SI were increased in structured versus unstructured transcribed regions (Fig. 5C,G,K; Supplemental Fig. S12). To account for this potential confounder, we ran enrichment tests for different ranges of GC content and phastCons score and observed the following (*P* < 0.05, one-sided Mann–Whitney *U* test, BH-corrected) (Supplemental Table S6): Expression of eRNAs and 3′ extensions overlapping CRSs with GC content between 25% and 75% is significantly increased in most tissues, whereas TSS-upstream (sense and anti-sense) transcription is rarely linked to CRS overlap. The data show that conserved structure predictions add significant information for distinguishing transcriptionally active regulatory sites from silent ones despite the impact of GC content and sequence conservation.

The positive correlation of CRSs and expression might be due to enhanced transcription and/or increased stability (slower degradation). To disambiguate these alternatives, we examined CAGE data for transcription initiation at DHSs in control versus exosome-depleted HeLa cells (Andersson et al. 2014b). Defining “stability” based on the change of expression level after exosome depletion (Methods), we find that stable eRNAs of preferentially unidirectional transcription were enriched for transcripts containing CRS regions (*P* = 0.002, FET, BH-corrected) (Fig. 5D). (We cannot rule out some impact of GC content and sequence conservation since the small sample size precludes GC-content-specific enrichment tests.) An example of a unidirectional stable eRNA is depicted in Figure 6D. The relationship between transcript stability and CRS presence in eRNAs may point to RNA structure alone as a contributor to stability, but the association of CRSs to stable TSS-upstream transcripts was not significant (Fig. 5H), implying that structure per se does not confer a stability advantage. We also note that thousands of CRS regions overlap RBP binding sites in promoter and enhancer regions (Supplemental Fig. S13), raising the possibility that CRSs mediate transcript stability by protein recruitment.

## Discussion

Although the RNA structure landscape is recognized as an important feature of the transcriptome, a global analysis of its functional impact in vertebrates is still missing. In our study, we present a comprehensive screen for CRSs based on local structural alignments of human and other vertebrate genomes. In general and in agreement with observations by Torarinsson et al. (2008), our approach substantially realigned many sets of orthologous sequences, exposing well-defined structures with lower SI than the input, but phylogenetically broader scope than was visible using earlier purely sequence-based approaches. Of the 774,000 CRSs covering 516,000 contiguous genomic regions, 276,000 showed an average pairwise SI below 60% over the 17 representative genomes in the phylogenetic tree. The CRSs were enriched in a spectrum of known functional elements, supporting their global functional importance. Clustering CRSs may help identify and dissect common RNA structures shared across multiple classes of RNAs (Parker et al. 2011; Miladi et al. 2017) that may have common functionality, such as specific protein–RNA binding sites.

Most CRSs may be weakly or narrowly expressed, hence not easily detectable by ordinary transcriptome-wide RNA-seq. We designed a custom capture chip to study them further and found 8385 CRS regions expressed in human fetal brain of which 662 were not in the publicly available brain data sets that we examined. The qRT-PCR and in vitro structure probing in human and mouse of a small but diverse subset of CRS regions revealed tissue-specific expression profiles and conserved secondary structures. These results support both our in silico predictions and CaptureSeq strategy for de novo discovery of structured RNA. Overall, our CaptureSeq detected expression for >10% of the approximately 77,000 probed CRS regions, of which ∼1% were novel expressed CRSs in just a single tissue, human fetal brain, demonstrating the concept. Our CaptureSeq strategy aims to detect novel RNAs on a transcriptome-wide scale. Similar approaches that tile entire genes (Mercer et al. 2014) are nicely complementary, as subsequent tiling around newly discovered expressed CRSs would meaningfully extend our knowledge about these transcripts.

We found CRS regions to significantly overlap functional elements, such as binding sites for RBPs (102,000), eRNAs (15,000), extended 3′ ends (8000), and extended 5′ ends (8000). Widespread enhancer- and TSS-upstream-anti-sense transcription are a still-recent observation from high-throughput sequencing; whether these transcripts have functional roles or are just noise remains controversial. Sometimes these transcripts produce lncRNAs that may contribute to the regulatory function of the genomic site (Rinn and Chang 2012). One crucial role of bidirectionally transcribed promoters and enhancers may be in transcript stabilization (Goodarzi et al. 2012). This process is relatively independent from the primary sequence and instead may be linked to RNA structure, e.g., through protein binding. Based on our analyses we conclude that (1) CRSs are associated with increased stability in certain genomic contexts, which at least partially explains higher abundance of structured elements seen in RNA-seq, but (2) the more general observation of greater abundance of structured elements (for a large range of GC contents) suggests that CRSs have functional roles above and beyond modulating stability.

In further support for functional roles of the CRSs, we observed that a substantial fraction of CRSs (∼36% of the 433,000 tested) were coexpressed in human and mouse, including CRSs in transcribed regulatory regions. Although the fraction of CRSs coexpressed in corresponding human and mouse tissues was low, it was significant for CRSs of several biotypes. Furthermore, it is likely that we underestimated this overlap because of low expression levels, differences in the biological material (tissues), and cross-platform differences between the experiments, which complicate all cross-species expression analyses.

The presence of structured RNAs of relatively low sequence conservation in lncRNAs (Fig. 1F) agrees with the observation that lncRNAs appeared recently in evolution (Rands et al. 2014; Washietl et al. 2014). A similar search, e.g., by FOLDALIGN (Sundfeld et al. 2016), in primates or other closely related species will likely elucidate more novel CRSs in lncRNA. Lower CRS sequence identities than in lncRNAs were observed in intergenic regions with signatures specific for active regulatory elements, supporting the idea that such structures play a role in ongoing evolution of transcriptional regulation.

Our computational screen complements large-scale experimental efforts to probe for RNA structures (Rouskin et al. 2014; Wan et al. 2014). These experimental approaches are limited to elucidating the structure propensity of single nucleotides and do not provide evidence for the base interaction map. Base interactions in human and mouse could potentially complement existing sequence alignments to build a base-interactome map (Lu et al. 2016). The substantial fraction of long-range interaction from such experiments could complement the short-range interactions from CRSs and thus together provide a more complete picture of the RNA structurome.

To conclude, our CRS screen is to our knowledge the first genome-wide screen in vertebrates explicitly based on local structural alignments that does not make rigid use of pregenerated sequence-based alignments. In combination with CaptureSeq, it has revealed RNAs not detectable by standard RNA-seq experiments and has the potential to reveal many more when repeated in other tissues and biological conditions. The CRSs themselves show evidence for purifying selection and colocalize with a range of known functional elements, especially in enhancers and near annotated gene boundaries. Similarly, we found CRSs overlapping numerous RBP binding sites for which RNA structures have not previously been reported. Thus, our study provides support for the existence and widespread functional importance of a broad landscape of novel RNA structure candidates widely conserved in vertebrates. Fully elucidating their roles will entail significant follow-on work.

## Methods

### Genome-wide screen for CRSs

CMfinder (Yao et al. 2006) locally aligns, folds, and describes predicted CRSs through covariance models using an expectation-maximization style learning procedure. To predict CRSs anchored in the human genome, hg38, we carried out the following steps: (1) filtered human-based 17-species MA from UCSC (http://hgdownload.cse.ucsc.edu/goldenPath/hg18/multiz17way) for length ≥60-bp blocks containing human and three or more nonprimates, resulting in 8,131,488 MA blocks covering 46% of the human genome; (2) realigned and predicted shared structure in these blocks using CMfinder (version 0.2.1 and 0.3 for pscore calculation for the vertebrate tree) with default parameters (maximal base-pair span of 100 bp: -M 100) in both reading directions; (3) used UCSC liftOver (http://hgdownload-test.soe.ucsc.edu/goldenPath/hg38/liftOver/) across genome builds to map coordinates of high-scoring (see next section) human CRSs from hg18 to hg38; (4) used liftOver across species to map human hg38 coordinates to orthologous regions in each of the other 99 vertebrate genomes in UCSC's 100-species alignment; (5) searched each such sequence (extended 50 bp up- and downstream) for hits using the CRS covariance models with Infernal cmsearch (Nawrocki and Eddy 2013); and (6) aligned these hits to the CRS covariance model with Infernal cmalign. Step 2 of the screen alone took >150 CPU years on a Linux cluster (each node with two Intel Xeon E5649 2.53 GHz - Westmere -EP and 24 GB memory). See Supplemental Methods for our rationale in choosing this strategy.

### Scoring scheme

The probabilistic ranking statistic, pscore, extends the phylo-SCFG approach of EvoFold (Pedersen et al. 2006). Like EvoFold, it contains both a single-nucleotide model (a general time-reversible model of sequence evolution on the four RNA bases) and a structured RNA model (analogous, on the 16 potential base pairs). A third model, also single nucleotide, captures poorly conserved (neutral or misaligned) regions. We predict structures where the structured RNA model outscores both unstructured alternatives. (Vertebrate genomes are heterogeneous mixtures of well- and poorly conserved regions. Including the third model avoids many potential false positives where poorly conserved regions score poorly, but by chance the structured model happens to outscore the unstructured-but-conserved model.) We also significantly revised EvoFold's parameterization of noncanonical base pairs, added a quasi-stationary model of base-pair indels and other gaps, and reduced the number of free parameters (22 versus EvoFold's 32). Parameters were trained on structure-annotated alignments (Wang et al. 2007). All three models are scored by maximum likelihood (Felsenstein 1981) on the 17-species vertebrate tree learned by phastCons (Siepel et al. 2005). Incorporation of folding energy is the final significant departure from EvoFold. Weighting SCFG posteriors by the thermodynamic partition function emphasizes covarying columns in structurally stable versus unstable contexts. Overall, this approach decreases the GC content bias seen in Torarinsson et al. (2008) and sharply reduces estimated FDR compared to that approach, to RNAz, and to EvoFold, as seen in the benchmarks reported in Yao (2008), chapter 4. See also Supplemental Methods.

### Mapping of genome-wide structure probing

We retrieved the data series GSE45803 (Rouskin et al. 2014) from the NCBI Gene Expression Omnibus (GEO), trimmed adapters and low-quality 3′ ends (Phred33 < 20) using the FASTX-toolkit (http://hannonlab.cshl.edu/fastx_toolkit/), and filtered for length ≥20 nt and average Phred33 > 20. Preprocessed reads were mapped to hg38 using BWA-MEM (Li 2013), disallowing 5′ soft-trimming (bwa mem -L 10000,5). The number of mapped reads initiating 1 nucleotide (nt) 3′ of each base position of the reference was calculated for the respective termination assays of the dimethyl sulfate (DMS) reaction of native RNA and denatured RNA. Read counts were normalized to sequencing depth, and the log-fold change was calculated using a pseudo-count of five to regularize low coverage regions: [log_2_(denature+5) − log_2_(native+5)]. Positions displaying a log-fold change larger than one were considered to be paired nucleotides. The CRS consensus structures were evaluated at nucleotide resolution using this genome-wide structure probing as a gold standard. See Supplemental Methods.

### Annotation enrichment corrected for SI and GC content

Statistical significance tests of CRS (CRS region) enrichment for genomic features reflect only the part of the genome corresponding to the MA input set and reflect careful control for GC content and SI. A normal approximation to the binomial distribution (one-sided *Z*-test, BH-corrected, “pnorm” function in R) (R Core Team 2016), *N*[μ = *np*, σ^2^ = *np*(1 − *p*)], was used to estimate a *P*-value based on the observed overlap count *q* (between middle coordinate of CRS and genomic feature, ignoring strand information), where *p* is the probability that a CRS overlaps a feature and *n* is the number of CRSs. The statistic was only calculated if the genome (bin) covered by the feature totaled at least 1 kb. We studied the CRS enrichment binned by GC content and by SI (denominator is gap-included alignment length; or phastCons) where each was calculated for 100-bp windows of concatenated MA blocks. Enrichment tests were repeated for CRSs filtered to remove repeat and (semi-)inverted repeat sequences resulting in the same conclusions.

### Evolutionary selection analysis

Selection in CRSs was tested in three ways. First, we estimated pairwise base distance (Ponjavic et al. 2007) between human, mouse, and macaque sequences using baseml (http://abacus.gene.ucl.ac.uk/software/paml.html) with model REV/GTR for CRSs (*d*_CRS_), ARs (*d*_AR_), and intergenic loci (*d*_Inter_) after removing gap columns in CRS alignments and 17-species MAs (ARs, intergenic). Purifying selection was defined as both selection ratios *d*_CRS_/*d*_AR_ and *d*_CRS_/*d*_Inter_ being smaller than 0.95 (15,131 CRSs). Second, CRSs enrichment inside indel-purified segments (IPSs) (Lunter et al. 2006) was conducted as described in Methods section “Annotation Enrichment Corrected for SI and GC Content.” Third, using whole-genome sequencing data from phase 3 of the 1000 Genome Project (The 1000 Genomes Project Consortium 2015), we analyzed the MAFs of polymorphisms in CRSs (low coverage .vcf from ftp://ftp.1000genomes.ebi.ac.uk/vol1/ftp/release/20130502/). See Supplemental Methods and Supplemental Figure S5.

### CLIP-seq analysis

CLIP data for 67 human RBPs and 10 mouse orthologs were collected from public databases (Supplemental Table S7). Reads were preprocessed using cutadapt v1.2.1 (Martin 2011) and mapped to hg38 or mm10 with Bowtie 2 (Langmead and Salzberg 2012). PCR duplicates were removed using Picard v.197 (http://broadinstitute.github.io/picard/) and peaks called (*P* < 0.01) using Piranha v1.2 (Uren et al. 2012). Enrichment studies for CRSs in human RBP binding sites were conducted as described in Methods section “Annotation Enrichment Corrected for SI and GC content.” Enrichment for CRSs in human–mouse conserved RBP binding sites was tested using FET (contingency table: CRS conservation versus RBP binding site conservation). A mouse RBP binding site falling within 50 bp of the human counterpart after liftover from mm10 to hg38 was considered conserved.

### Expression analysis

Premapped reads (.bam) to the human genome (hg38) were analyzed from publicly available total RNA-seq libraries of 19 tissues (ENCODE phase 3 [The ENCODE Project Consortium 2012]) and poly(A) RNA-seq libraries of 16 tissues (Illumina Human Body Map 2.0 [HBM]). All libraries are listed in Supplemental Table S8. Uniquely mapped reads were counted for overlap with CRS regions (201-bp window around the center of CRS region) using featureCounts v1.5.0-p1 (parameters -s 0 -T 8 -Q 10 -p -P -d 50 -D 300 -C -B --read2pos 5) (Liao et al. 2014). To define library-specific cutoffs for expression, we calculated an empirical *P*-value based on the read count distribution of random genomic loci (201-bp window) from the MA input set. Regions whose read counts have *P* < 0.01 were considered to be expressed. Read counts were converted to count per million mapped reads (CPM) and normalized between libraries using the relative log expression (RLE) normalization procedure in edgeR (Robinson et al. 2010). In case of replicates, we calculated mean values for normalized read counts for each tissue.

### CaptureSeq experiment

We designed 125,000 probes, each 60 bp long, covering both strands, more than three mismatches to their closest genomic paralog, representing 77,320 CRS regions with largest pscores and conservation between human and mouse. More than 70% of probed CRS regions were intronic or intergenic. Total RNA from human fetal brain (Clontech) was DNase I treated (Invitrogen), rRNA was depleted using RiboMinus (Invitrogen) according to the manufacturer's recommendations, cleaned on Microcon YM-30 columns (Millipore), chemically fragmented, and cleaned on Microcon YM-10 columns (Millipore). Fragmented RNA was reverse transcribed using a random hexamer with an attached adaptor. Following reverse transcription, second-strand synthesis was performed, blunt ended, and adaptor ligated. The library was size-selected; 100–200 bp were excised and cleaned. Excised fragments were enriched by 18 cycles of PCR and cleaned (Qiagen). The library was split into two equally sized sublibraries for investigating two different annealing temperatures to optimize the hybridization step of potentially self-folded RNA probes. The dried library was mixed with hybridization buffer and denatured, immediately loaded onto the custom chip (NimbleGen), and incubated for 3 d at 70°C. The slide was eluted according to the manufacturer's protocol (NimbleGen Arrays User's Guide, sequence capture array delivery, version 3.2). Following elution, the samples were enriched by 19 cycles of PCR and cleaned (Qiagen). The chip was stripped and rehybridized with the second half of the library for 3 d at 42°C. The eluate was washed and enriched as described above.

### Analysis of CaptureSeq

Reads (.fastq) were trimmed for low-quality 3′ ends (Phred33 < 30) and adapters, and trimmed reads shorter than 40 nt were discarded using cutadapt v1.8.3. Cleaned reads were aligned to the human genome (hg38) using STAR 2.5.2a (default parameters) (Dobin et al. 2013) and alignments of ≤2 mismatches were reported (--outFilterMismatchNoverLmax 0.03). The following strategy defined read islands, their read counts, and significance of RNA probe-assigned read islands (on-targets): (1) filter uniquely mapped reads and remove simple repeats (≥50% overlap); (2) extend reads to 150 nt in reading direction (unified reads) and define a region of overlapping unified reads as a read island; (3) count reads inside a read island; (4) intersect read islands with RNA probes (overlap ≥1 nt); and (5) calculate empirical *P*-value for read counts of RNA probe-assigned read islands. The empirical *P*-value is based on the read count distribution of off-targets (read islands that are not assigned to RNA probes), and we selected *P* < 0.1 for expressed RNA probes (Supplemental Fig. S8A).

### qRT-PCR

The tissue-specific expression profiles of 23 selected CRS regions (CaptureSeq *P* < 0.1) were determined by qRT-PCR using purified total RNA from seven different tissues (brain, colon, heart, kidney, liver, small intestines, and testis) in human and mouse. Human total RNA from these seven tissues (plus fetal brain) were ordered (Clontech). The same tissues were isolated from 30-d-old male mice (Balbc/J), and total RNA was extracted using a modified miRNeasy protocol (Qiagen). See Supplemental Methods.

### RNA structure probing

CRS sequences from human and mouse were selected for structure probing based on low FDRs, low SI between human and mouse, and expression in human and mouse brain as determined by qRT-PCR. Templates were made by PCR on gDNA templates and designed to include flanking sequences to the extent that it would facilitate the predicted structures upon folding (Hecker et al. 2015). CRS in vitro transcripts were screened by native gel electrophoresis, and pairs that yielded single bands were subjected to structure probing using RNase V1 and S1 nuclease or Pb^2+^ for demonstration of double- and single-stranded regions, respectively. See Supplemental Methods.

### Definition of gene regulatory regions

Initially, we defined enhancers through ENCODE chromatin segmentation states (classes E or WE in ≥2 of 6 human cell lines; length 100 bp to 1 kb) (Hoffman et al. 2013), loci upstream (−2 kb to −100 bp) of TSSs, and loci downstream (+1 bp to +2 kb) of 3′ ends of mRNAs and lncRNAs annotated in GENCODE. We considered only genes of >10 kb away from their adjacent annotated genes (∼13,000 genes). For more stringent definition of regulatory regions, taking experimentally measured transcript boundaries into account, we used the following data: DNase I hypersensitivity peak clusters from ENCODE (95 cell types) (The ENCODE Project Consortium 2009), CAGE expression of robust (>10 TPM) peaks (length ≤200 bp) from FANTOM5 Phase 2.0 (Andersson et al. 2014a), ENCODE chromatin segmentation states, GENCODE gene/TSS annotation of mRNAs and lncRNAs, and polyadenylation signals (Gruber et al. 2016). TSS upstream transcription and enhancers were defined by CAGE peaks, DHSs, and chromatin states. Alternative 3′ ends were defined by poly(A) signals. See Supplemental Material and Supplemental Figure S11.

### Expression of structured and unstructured gene regulatory regions

Differences in expression level in tissues between structured (ones overlapping CRSs) and unstructured regulatory regions in different GC content or phastCons score bins were tested by one-sided Mann–Whitney *U* test, BH-corrected, and considered significant if *P* < 0.05 in all replicates. Uniquely mapped reads from ENCODE phase 3 and E-MTAB-513 (Supplemental Table S8) were counted for overlap with regulatory regions using featureCounts v1.5.0-p1 (parameters -s 0 -T 8 -Q 10 -p -P -d 50 -D 2000 -C -B --read2pos 5). Read counts were normalized to CPM/RLE before hypothesis testing. GC content and phastCons were measured from regulatory regions.

### Transcript stability

To test for stability of transcripts, we analyzed triplicate published CAGE libraries from hRRP40 (exosome) depleted and EGFP depleted (control) HeLa cells (Andersson et al. 2014b). We got premapped 5′ ends of sequenced capped RNAs for all six libraries from the investigators (.bed). Exosome sensitivity was calculated as described previously (Andersson et al. 2014b) for both strands by max[(*E*_*exo*_ − *E*_*ctr*_)/*E*_*exo*_, 0], with *E*_*exo*_ and *E*_*ctr*_ denoting the expression level after exosome depletion and in control HeLa cells, respectively. We used thresholds of ≤0.25 and ≥0.75 to identify highly stable and highly unstable RNAs emanating from transcribed DHSs. See Supplemental Methods.

### Statistics and visualization

Statistical analyses and visualization were performed with R (R Core Team 2016), feature distances were calculated using BEDTools (Quinlan and Hall 2010), genomic views were from UCSC Genome Browser (Rosenbloom et al. 2015), and structures/alignments were drawn with Vienna RNA Package tools (Lorenz et al. 2011).

## Data access

The CaptureSeq data from this study have been submitted to the NCBI Gene Expression Omnibus (GEO; http://www.ncbi.nlm.nih.gov/geo/) under accession number GSE87214. The list of CRSs, CRS alignments, CRS annotation, and on-target expressed (*P* < 0.1) regions found by CaptureSeq are provided as Supplemental Data. Our catalog of predicted CRSs, Supplemental Data, and a UCSC track hub of CRSs are available at http://rth.dk/resources/rnannotator/crs/vert.

## Supplementary Material

Supplemental Material
