# Supplementary material for: The identification and functional annotation of RNA structures conserved in vertebrates
Source: Genome Res. 2017 Aug;27(8):1371–83. doi: 10.1101/gr.208652.116 (PMC5538553; doi:10.1101/gr.208652.116)
Supplement: Supplemental Material [file supp_27_8_1371__index.html]

The identification and functional annotation of RNA structures conserved in vertebrates — Supplemental Material 

# The identification and functional annotation of RNA structures conserved in vertebrates

## Supplemental Material

- Supplemental\_Methods.pdf
- Supplemental\_Data\_1.csv.gz
- Supplemental\_Data\_2.csv.gz
- Supplemental\_Data\_3.csv.gz
- Supplemental\_Data\_4.csv.gz
- Supplemental\_Fig\_S1.pdf
- Supplemental\_Fig\_S2.pdf
- Supplemental\_Fig\_S3.pdf
- Supplemental\_Fig\_S4.pdf
- Supplemental\_Fig\_S5.pdf
- Supplemental\_Fig\_S6.pdf
- Supplemental\_Fig\_S7.pdf
- Supplemental\_Fig\_S8.pdf
- Supplemental\_Fig\_S9.pdf
- Supplemental\_Fig\_S10.pdf
- Supplemental\_Fig\_S11.pdf
- Supplemental\_Fig\_S12.pdf
- Supplemental\_Fig\_S13.pdf
- Supplemental\_Table\_S1.pdf
- Supplemental\_Table\_S2.pdf
- Supplemental\_Table\_S3.pdf
- Supplemental\_Table\_S4.pdf
- Supplemental\_Table\_S5.pdf
- Supplemental\_Table\_S6.pdf
- Supplemental\_Table\_S7.pdf
- Supplemental\_Table\_S8.pdf
- Supplemental\_Table\_S9.pdf
- Supplemental\_Table\_S10.pdf
- Supplemental\_Table\_S11.pdf
