# Supplementary material for: The identification and functional annotation of RNA structures conserved in vertebrates
Source: Genome Res. 2017 Aug;27(8):1371–83. doi: 10.1101/gr.208652.116 (PMC5538553; doi:10.1101/gr.208652.116)
Supplement: Supplemental Material [file supp_gr.208652.116_Supplemental_Methods.pdf]

The identification and functional annotation of RNA structures conserved in vertebrates

Stefan E Seemann, Aashiq H Mirza, Claus Hansen, Claus H Bang-Berthelsen, Christian Garde, Mikkel Christensen-Dalsgaard, Elfar Torarinsson, Zizhen Yao, Christopher T Workman, Flemming Pociot, Henrik Nielsen, Niels Tommerup, Walter L Ruzzo, Jan Gorodkin

SUPPLEMENTAL METHODS

1. Genome-wide screen for CRSs
2. CMfinder scoring scheme
3. *In silico* false discovery rate estimation
4. Mapping of genome-wide structure probing
5. CRS annotation
6. Evolutionary selection within CRSs
7. Comparison of expression in human and mouse
8. qRT-PCR study
9. RNA structure probing
10. Definition of gene regulatory regions
11. Transcript stability

1. Genome-wide screen for CRSs

The strategy of a genome-wide screen for RNA structural alignments with an initial hg18-based 17-species MULTIZ alignment, as described in the Methods, was chosen because the running time of CMfinder prevents an initial screen on all 100 species, and alignment blocks in the hg38-based 100-species MULTIZ alignment with length ≥60bp and ≥ 3 non-primate species cover only 22% of the human genome. The 17-species vertebrate tree is a reasonable representation of a redundancy reduced version of the 100-species vertebrate tree. The re-alignment percentage was calculated as described in Torarinsson et al. (2008) and considered only non-gapped positions in human. Comparing CMfinder's hg18-based 17-species alignments and corresponding 17-species subalignments extracted from the hg38-based 100-species versions built as described in the Methods, only 1.1% of positions were realigned, strongly demonstrating the robustness of our alignment methodology.

2. CMfinder scoring scheme

As summarized in the doctoral dissertation "Genome scale search of noncoding RNAs: bacteria to vertebrates" of Zizhen Yao (University of Washington Seattle, 2008), pscore uses a phylogenetic stochastic context free grammar (phylo-SCFG)-based probabilistic model to measure the statistical confidence of predicted RNA structures. The phylo-SCFG model combines the phylogenetic model's ability to describe evolutionary history, and the SCFG's ability to model RNA secondary structure, and has previously been used in Evofold, Pfold, and other studies. Although Evofold has been demonstrated to perform well on conserved alignments, we found it inappropriate for scoring `CMfinder` structures, many of which have low sequence similarity. In particular, `CMfinder` sometimes finds structural motifs with a stable hairpin within unrelated sequences. Evofold tends to give very significant scores for such structures, which we believe is due to "one size fits all" model for the genomic sequences, which in practice are a heterogeneous mixture of well- and poorly conserved segments. To address this weakness, we add a third model to describe poorly conserved regions that are under neutral selection or are due to alignment errors. The basic idea underlying our approach is that we test a given alignment under three competing hypotheses: it is a non-conserved region, a sequence conserved region or a structurally conserved region. This approach predicts a structural alignment as a functional RNA if the alignment favors the structural RNA model significantly against both alternatives.

On the phylo-SCFG side, we explicitly model the transitions between conserved and non-conserved modes within single-stranded regions to capture the typical mosaic conservation pattern in RNA structures that resembles the phlyo-HMM used in phastCons. Our evolutionary tree model follows the general probabilistic framework of Evofold, which includes an evolutionary tree, substitution rate matrices for single-stranded regions and double-stranded regions, and corresponding vector for equilibrium frequencies. For the double-stranded evolutionary model, we used a reduced-parameter setting based on whether the base pairs are canonical or not for a more parsimonious representation. We also introduced a special gap model that favors a single base pair deletion event over two consecutive events of losing one nucleotide on each strands. This model maintains relatively stable equilibrium frequency of nongap characters, while the gap frequency increases with time.

We train our phylo-SCFG parameters by a maximum likelihood approach. Given a set of structurally annotated alignments, we try to estimate parameter values so that the likelihood of the alignment given the model is maximized. Given the structure annotation, the maximum likelihood estimation of the grammar is independent of the alignment and the evolutionary model, and the estimation

of the evolutionary model only relies on the alignment and the structure. Therefore, grammar and evolutionary model can be estimated separately. The evolutionary tree we used for is the species tree learned by phastCons [1] based on the 17-way MULTIZ alignments. To obtain a set of structural alignments of homologous ncRNAs, we collected the data from the study by Wang *et al.* [2], which for each seed Rfam member in human, includes matches of the corresponding Rfam Covariance model in all multiple alignment blocks within 10K range of the human member. We took these Rfam CM matches, and aligned them to the corresponding CM model using the "cmalign" method in the Infernal package. If multiple CM matches are found in one species, we chose the one with the highest sequence identity to the human seed member. We produced in total 264 structurally annotated alignments. Since our production rules are not ambiguous, their probabilities can be calculated directly from parsing of the structural alignments. The maximum likelihood estimates of evolutionary model parameters were found using the BFGS quasi-Newton algorithm.

We investigated two scoring schemes. First, we used the negative log probability that there is no structured region in the alignment. This scoring scheme considers all possible structures that contribute to the probability that the alignment contains a structured region. We refer to this score as the totRNA score. To evaluate the quality of a given structural annotation, we score each annotated base pair by $\log(1 - P(\text{pair}(i, j)))$, where $P(\text{pair}(i, j))$ is the posterior probability that columns i, j are paired by summing over all parses that emit a pair at i, j. The score for an overall structure is the sum of scores for all its base pairs. Note that this score does not correspond to any probability, because the posterior probabilities of base pairs are not independent. Compared to the alternative using the posterior probability of the given whole structure, this score is robust to partial prediction errors: if a predicted base pair has very low posterior probability, its score is near zero and ignored. The overall score is dominated by a set of high quality base pairs. We refer to this score as the pair score (pscore). Occasionally, some pairs of columns share great covariation by chance, without support from corresponding folding energy. Such pairs may still receive significant posterior probabilities. To take this issue into account, we multiply the emission probabilities of base paired columns by the values of corresponding partition function [3]. We define the partition function for a pair of columns as the geometric mean of the partition functions of all sequences at given positions. In our investigations, pscore was more robust, and was used throughout our paper.

3. *In silico* false discovery rate estimation

In silico FDR of a specific pscore threshold τ estimates discrimination of real structures (R_{real}) from ones occurring by chance ($\text{R}_{\text{simulated}}$):

$$\text{FDR}(\tau) = \frac{\text{FP}(\tau)}{\text{FP}(\tau) + \text{TP}(\tau)} \sim \frac{\text{R}_{\text{simulated}}(\tau)}{\text{R}_{\text{real}}(\tau)}.$$

False positives (FP) were estimated by generating dinucleotide-controlled alignments ($\text{R}_{\text{simulated}}$) using SISSIz [4] (parameters –simulate –tstv –rna –maf) for a random 10% of the human-based (hg18) 17-species MULTIZ alignment. Despite pscore's careful modeling of evolutionary conservation, empirical scores from dinucleotide-controlled null sequences showed that it was not entirely neutral to the broad range of GC contents seen in vertebrate genomes. Given the well-known association of GC content to various functionally relevant genomic features, we explicitly controlled for it by separately estimating the FDR in different GC-content bins. See Supplemental Fig. S1.

4. Mapping of genome-wide structure probing

Our experimental approach to assess the prediction performance of the genome-wide screen for CRSs is based on dimethyl sulphate (DMS) modifications of unpaired residues, followed by deep sequencing. For details on the experimental procedure and conditions, we refer to Rouskin *et al.* [5]. The DMS induced termination of the reverse-transcriptase during library preparation enables the identification of modified residues on a transcriptome-wide scale. A propensity for paired nucleotides was derived by comparing the DMS termination of a sample of native RNA to that of a sample of denatured RNA. Specifically, this was achieved by first counting the number of mapped reads initiating at the 3' neighboring nucleotide for each base position of the reference genome for the respective assays ($\alpha_i(p)$). These positions were filtered to adenine and cytosine bases which can be affected DMS. A normalization factor ($\Omega_{i,j}$) was computed to account for differences in sequencing depths. Subsequently, the structure propensity ($\Gamma_i(p)$) was calculated as the log-fold change in read counts between the denatured and the native samples of RNA at every base position. A pseudo count of 5 was introduced to regularize low coverage positions, i.e. only well probed positions will be considered downstream in the analysis. Positions with log-fold changes larger than 1.5 were considered paired nucleotides, and an equally large set of positions were sampled from those with the lowest log-fold changes and were considered unpaired nucleotides.

$$\Gamma_{vivo}(p) = \log_2(\alpha_{denature}(p) + 5) - \log_2(\Omega_{vivo} \cdot \alpha_{vivo}(p) + 5) \tag{1}$$

$$\Gamma_{vitro}(p) = \log_2(\alpha_{denature}(p) + 5) - \log_2(\Omega_{vitro} \cdot \alpha_{vitro}(p) + 5) \tag{2}$$

$$\Omega_i = \sum_p \alpha_{denature}(p) / \sum_p \alpha_i(p), \quad i \in \{vivo, vitro\} \tag{3}$$

When paired and unpaired positions have been defined, the genomic overlap with the CRS consensus structure was determined and used to derive the contingency table by assigning paired nucleotides as positives and unpaired nucleotides as negatives. For an overview we refer to Supplemental Fig. S2A.

The genome-wide structure probings were conducted in *in vivo* and *in vitro* experimental conditions. The contingency tables are depicted in Supplemental Fig. S2B for the two validation sets. We used the following metrics to assess the performance: sensitivity, specificity, positive predictive value, Mathews correlation coefficient, and the odds-ratio, which are defined as follows:

$$\text{Sensitivity} = \frac{\text{TP}}{\text{TP} + \text{FN}} \tag{4}$$

$$\text{Specificity} = \frac{\text{TN}}{\text{FP} + \text{TN}} \tag{5}$$

$$\text{Positive Predictive Value} = \frac{\text{TP}}{\text{TP} + \text{FP}} \tag{6}$$

$$\text{Mathews Correlation Coefficient} = \frac{\text{TP} \cdot \text{TN} - \text{FP} \cdot \text{FN}}{\sqrt{(\text{TP} + \text{FP})(\text{TP} + \text{FN})(\text{TN} + \text{FP})(\text{TN} + \text{FN})}} \tag{7}$$

$$\text{Odds-ratio} = \frac{\text{TP} \cdot \text{TN}}{\text{FP} \cdot \text{FN}} \tag{8}$$

The performance is reported in Supplemental Fig. S2G for all studied CRSs and the top 20% fraction (when ranked according to confidence). The (Matthews) correlation coefficient was relatively low with 0.38 and 0.26 for the *in vivo* and *in vitro* condition respectively, which was, however, not surprising since CMfinder predicted base pairs require support from conservation. An improvement of the performance was gained when restraining the CRSs to the top 20% fraction. The reason that this

improvement might seem modest is that the distribution of CRS confidence scores is heavily tailed at the low end, hence the top 20% cutoff is pscore= 72.4 (data not shown).

The *in silico* false discovery rates of CRSs decrease with increasing CRS pscores (Supplemental Fig. S1). Similarly, we assessed the correlation between performance and pscores with the genome-wide structure probing by stratifying the CRSs according to their pscore and evaluating the performance for each stratum, see Supplemental Fig. S2C-F. The genome-wide structure probings show the same trend with increasing positive predictive values for increasing pscores.

5. CRS annotation

We used an adaptation of RNAnnotator [6] to human and mouse to annotate ncRNAs. If annotations were not available for assemblies hg38 and mm10, genomic coordinates were converted using UCSC liftOver. Intersections and distances of genomic features were calculated using BEDTools [7]. If not stated differently, CRSs (CRS regions) were considered mapped to features if either $\geq 50\%$ of the CRS (CRS region) length or $\geq 50\%$ of the feature length was covered (without strand consideration).

We used the following sources for gene annotation: GENCODE v25 and M10 [8], Rfam 12.1 [9], mirBase 21 [10], snoRNABase 3 [11] and lncRNAs from RNA-seq data (PLAR) [12]. Biotype annotation was defined in the following order: mRNA exons (UTR and CDS), lncRNA exons, sncRNAs, 2kb upstream of mRNA/lncRNA TSSs (5' extension), 2kb downstream of mRNA/lncRNA 3' end (3' extension), human ENCODE chromatin segmentation state of enhancers (classes E or WE) [13], intronic of mRNA/lncRNA, intergenic. TF binding sites of 161 factors were from ChIP-seq experiments from the ENCODE project [14]. Splice sites were from human exon-intron junctions in GenBank and alternative splicing events in UCSC genes.

6. Evolutionary selection within CRSs

Selection in CRSs was tested by their selection ratios, enrichment in indel-purified segments, and their minor allele frequencies. Here, two of these tests are described in detail.

Selection ratio based on Ancestral Repeats

We analyzed ratios of base distances between primates (human vs rhesus macaque) and to rodents (human vs mouse) of CRSs and local ancestral repeats (ARs) [15]. To find ARs within 1kb of CRSs, we searched for human repeats (RepeatMasker v3.2.9) expected to be present in mouse, hence, "ancestral" with respect to a common ancestor. We tested 26,224 CRSs with length $\geq$100 nt, conservation in human, mouse and macaque, adjacent conserved ARs, and no overlap to repeats, mRNAs (GENCODE v12) or indel-purified segments (IPSs) [16]. We randomly selected intergenic loci 1 to 2kb away from CRSs. The selection ratios based on ARs and local intergenic loci were suppressed in CRSs ($d_{CRS}/d_{AR} < 0.95$ AND $d_{CRS}/d_{Inter} < 0.95$) which is a signature of purifying selection for a large fraction of measured CRSs.

The differences to the substitution rates of local ARs were even larger when we considered only CRSs at a minimum distance of 10kb from the nearest mRNA annotated in Gencode v12, RefSeq and UCSC. Median d_{CRS}/d_{AR} ratios for base distances were 0.392 (human-mouse) and 0.664 (human-rhemac). We got similar results when we considered as estimate of neutral evolution local intergenic loci. The median d_{Inter}/d_{AR} ratios were close to 1 showing that intergenic loci and ARs behave similar, hence,

are both good neutral models. The support for purifying selection can be generalized to all CRSs because CRSs adjacent to ARs (1kb distance) had significantly smaller sequence identities (SI) than CRSs without ARs ($P < 10^{-51}$, KS-test).

Selection ratio based on PhastCons

We repeated the AR study using phastCons scores [1] as alternatives to the base distance for identifying the selection ratio. PhastCons considers the genomic region around ARs and CRSs through a phylogenetic hidden Markov model of sequence evolution and, thus, is less driven by local features. We downloaded phastCons scores from `ftp://hgdownload.cse.ucsc.edu/goldenPath/hg18/phastCons17way/` and calculated the mean phastCons scores for the same sequences used above: ancestral repeats (ARs) with length of at least 100 bp between human and rodents inside 1kb distance of CRSs, the corresponding CRSs, and corresponding intergenic loci filtered for no IPSs, no repeats and minimum distance of 1kb to CRSs. We calculated the log odds ratios $\log(\text{phastCons}_{CRS}/\text{phastcons}_{AR})$, $\log(\text{phastCons}_{CRS}/\text{phastcons}_{Inter})$, and $\log(\text{phastCons}_{Inter}/\text{phastcons}_{AR})$ (Supplemental Fig. S5C). In agreement to the base distance between primates and rodents, we observed significantly larger phastCons scores for CRSs compared to ARs and local intergenic regions ($P = 0$, KS-test), whereas ARs and intergenic regions have comparable phastCons scores.

Minor allele frequencies

Using whole genome sequencing data from Phase 3 of the 1000 Genome Project for 2,504 individuals from 26 worldwide populations we tested for evidence of selection in CRSs by analysing their minor allele frequencies (MAFs). Similar to the study in [17], CRSs were only considered if they are at least 2 kb away from known protein-coding genes (Gencode v12 annotations; 271,247 CRSs). Low coverage variant call format (vcf) files were used to calculate allele frequency data across all populations (`ftp://ftp.1000genomes.ebi.ac.uk/vol1/ftp/release/20130502/`). The average MAF was calculated for CRS overlapping mutations in the 100 bp long loci around the middle coordinate of the CRS. To consider whether the decrease in average MAF around CRSs is significant, we tested whether the average MAF of CRSs is significantly different to the average MAFs in distal regions (in the 100 bp long windows spanning from 5 kb to 10 kb and from -5 kb to -10 kb, relative to the CRS, that do not overlap known mRNAs). We corrected for sequence identity (SI) by splitting the search space into 11 SI bins: (0,0.4], (0.4,0.45), (0.45,0.5), (0.5,0.55), (0.55,0.6), (0.6,0.65), (0.65,0.7), (0.7,0.75), (0.75,0.8), (0.8,0.85), and (0.85,1]. Amongst the tested CRSs we observed a significant decrease in average MAF for all chosen 11 SI bins of CRSs ($p<0.01$ for SI=(0,0.4], $p< 10^{-16}$ for the other 10 SI bins, Mann-Whitney U-test). In 16.5% of investigated CRS sequences was the minor allele frequency significantly smaller than in its distal regions (empirical rank-based P-value $p<0.01$).

7. Comparison of expression in human and mouse

Well-documented technical biases and the specificity of spatio-temporal expression patterns [18, 19] limited the availability of matching RNA-seq data sets between human and mouse to 10 tissues. Human and mouse total RNA-seq libraries of 4 tissues (heart, liver, diencephalon/forebrain and cerebellum/hindbrain) (ENCODE phase 3 [20]) were downloaded as premapped reads (.bam) and poly(A) RNA-seq libraries of 6 tissues (testis, liver, kidney, heart, cerebellum and brain) [21] as raw reads (.sra) (Supplemental Table S8). Raw reads were quality controlled with cutadapt version 1.8.3 (parameters -q 10 -m 40), and aligned to the genome (hg38 and mm10) with STAR 2.5.2a [22] (default

parameters). Feature counts, empirical P-value and normalization were calculated as described in the Methods section "Expression analysis". Random genomic loci had to be conserved in human and mouse in MULTIZ alignment input set. For RLE normalization human and mouse libraries were combined. The similarity of expression profiles between human and mouse for each CRS region was quantified using Pearson's correlation coefficient between vectors of normalized expression values of all tissues. For human-mouse shared biotypes of CRS regions we required the same annotation in both species, except for enhancers being exclusively defined in human.

8. qRT-PCR study

Human total RNA from Cerebellum (left and right respectively), Diencephalon, Frontal, Occipital, Parietal and Temporal lobes (BioCat, Heidelberg, Germany), adult and fetal brain, colon, pancreas, kidney, liver, heart, testis, small intestines were ordered (Clontech). Seven tissues (brain, kidney, liver, heart, testis, small intestines and colon) were isolated from 30 days old male mice (Balbc/J), and βTC-3 and αTC-1 cells were cultured [23]. The RNA extractions for both tissues and cells were performed using a modified miRNeasy protocol (Qiagen). For all samples, quality of RNA was assessed spectrophotometrically by NanoDrop (Thermo Scientific) and electrophoretically by Bioanalyzer (Agilent) and samples with RIN (RNA integrity number) values above 7 were used. RNA was treated with DNAseI (Qiagen), quantitated and reverse transcribed using iScript cDNA synthesis kit (BioRad) following manufacturer's instructions. Primers for 23 selected RNA regions were designed using Oligo Primer Analysis Software [24] for human and Primer3plus [25] for the mouse counterparts (Supplemental Table S9). All oligos were ordered from DNA technology and TAGC Copenhagen. For normalization primers were designed for 6 stable housekeeping genes from human and 3 from mouse (Supplemental Table S10). Stability of housekeeping genes were assessed using geNorm software [26]. cDNAs were subjected to qRT-PCR analysis using 20 ng cDNA as template in each reaction. All qRT-PCR reactions were run in triplicate. qRT-PCR was performed using Lightcycler Fast start DNA MasterPlus SYBR green I kit (Roche) or SYBR green master mix (Exiqon), essentially as described by the manufacturer, except that the reaction volume was decreased to 10 μl. qRT-PCR reactions were run on an Opticon2 thermocycler (BioRad) or an CFX384 thermocycler (Biorad). Following qRT-PCR, data was normalized using a normalization factor, calculated with geNorm, based on the described housekeeping genes.

9. RNA structure probing

A set of 10 pairs of `CMfinder` predicted CRSs from human and mouse were selected for structure probing based on high pscores and expression in human and mouse brain and colon as determined by qRT-PCR. A prototype of RNAcop [27] was used to define the extent of flanking sequences that preserve the predicted structures and allows analysis by primer extension. The primers for PCR and primer extension were designed using Primer3 [28] and are listed in Supplemental Table S11. Templates for in vitro transcription were made by PCR using Phusion DNA polymerase (Thermo Scientific) and human and mouse gDNA as templates. The PCR products were purified using the GeneJET PCR Purification Kit (Thermo Scientific) and in vitro transcribed into ^{32}P-labelled transcripts for folding analysis or un-labelled transcripts for structure probing using T7 RNA polymerase (Thermo Scientific) according to standard protocols. Purified transcripts were denatured by heating to 90°C for 1 min in 20 mM Tris-HCl pH 7.8, 140 mM KCl. Then, the transcripts were folded by incubation at 60°C for 15 min, slow-cooling to 30°C over a period of 15 min, addition of $MgCl_2$ to a final concentration of 3 mM, and further incubation at 30°C for 15 min. Renatured, radiolabeled transcripts were subjected to

native gel electrophoresis in 10% polyacrylamide gels (34 mM Tris-HCl pH 7.5, 66mM HEPES pH 7.5 and 3 mM $MgCl_2$) [29]. Pairs of transcripts in which both the human and mouse transcripts migrated as single bands (>90%) were selected for structure probing. Un-labelled transcripts were used for structure probing using RNase V1 for cleavage of double-stranded segments, and S1 nuclease or Pb^{2+} for cleavage of single-stranded regions [30][31]. The probes were titrated to single-hit conditions on each individual RNA. After cleavage, the RNA was subjected to primer extension using oligos labelled at the 5'-end with ^{32}P and M-MuLV reverse transcriptase (Thermo Scientific) [32]. The primer extension products were analysed by gel electrophoresis on 8% denaturing (50% urea) polyacrylamide gels along with sequencing ladders made by Sanger sequencing of the corresponding PCR-product with the same oligo as used for primer extension.

10. Definition of gene regulatory regions

A schematic illustration of the characterization of structured regulatory regions is provided as Supplemental Fig. S10. Regions around DNase hypersensitive sites (DHSs) were only considered if the distance from the DHS center to mRNA/lncRNA exons was at least 1kb, or all GENCODE annotated exons inside the 600bp window around DHS center were downstream of TSSs of mRNAs/lncRNAs on the plus strand or upstream of mRNAs/lncRNAs on the minus strand. For bidirectional transcription, the largest genomic coordinate of CAGE minus signal was smaller then the smallest genomic coordinate of CAGE plus signal in the 600bp window around DHSs. In the FANTOM5 CAGE data from 1,829 samples we found 4,307 loci with bidirectional transcription, 25,472 and 23,379 loci with unidirectional transcription on plus and minus strand, respectively. We defined regulatory regions as structured if any CRS overlapped (≥50%) downstream of a CAGE defined TSS inside the 2kb window around the DHS center. For poly(A) site definition we used experimental support of alternative 3' ends by polyadenylation signals as described by Gruber et al. (Gruber et al. 2016). We found 2,885 mRNAs and 1,260 lncRNAs with an alternative poly(A) site at least 50 bp downstream of the most distal GENCODE v25 annotated 3' end.

11. Transcript stability

Hg19 premapped 5' ends of sequenced capped RNAs for published CAGE libraries (triplicates) from hRRP40 depleted (exosome-depleted) and EGFP depleted (control) HeLa cells (Andersson et al. 2014b) were provided by Robin Andersson. We clustered adjacent CAGE tags on the same strand that are at most 20 bp apart after pooling all libraries. The expression of these tag clusters in each library was quantified by counting TPM and normalizing between libraries using RLE. For further analysis we kept only tag clusters with TPM/RLE≥1 in at least one replicate and selected the maximal TPM/RLE of control and exosome-depleted libraries as representatives. Finally, genomic coordinates were lifted over from hg19 to hg38. Exosome sensitivity was calculated as described in (Andersson et al. 2014b) for both strands by $\max((E_{exo}-E_{ctr})/E_{exo},0)$, with E_{exo} and E_{ctr} denoting the expression level after exosome (hRRP40) depletion and in control HeLa cells, respectively. We used thresholds of ≤0.25 and ≥0.75 to identify highly stable and highly unstable RNAs emanating from transcribed DHSs. Again, only tag clusters inside the 600bp window around DHS center were considered (see Supplemental Fig. S10). In the three control CAGE libraries we found 3,045 DHSs with bidirectional transcription, 5,712 and 5,646 DHSs with unidirectional transcription on plus and minus strand, respectively. Note that these data has relatively more bidirectional transcription compared to the FANTOM5 CAGE data that uses a more conservative definition of CAGE clusters.

SUPPLEMENTAL REFERENCES

References

[1] A Siepel, G Bejerano, J S Pedersen, A S Hinrichs, M Hou, K Rosenbloom, H Clawson, J Spieth, L W Hillier, S Richards, G M Weinstock, R K Wilson, R A Gibbs, W J Kent, W Miller, and D Haussler. Evolutionarily conserved elements in vertebrate, insect, worm, and yeast genomes. *Genome Res*, 15(8): 1034–1050, August 2005. doi: 10.1101/gr.3715005.

[2] A.X. Wang, W.L. Ruzzo, and M. Tompa. How accurately is ncRNA aligned within whole-genome multiple alignments? *BMC Bioinformatics*, 8:417, 2007. doi: 10.1186/1471-2105-8-417.

[3] J.S. McCaskill. The equilibrium partition function and base pair binding probabilities for RNA secondary structure. *Biopolymers*, 29(6-7):1105–1119, May 1990. doi: 10.1002/bip.360290621.

[4] T. Gesell and S. Washietl. Dinucleotide controlled null models for comparative RNA gene prediction. *BMC Bioinformatics*, 9:248, 2008. doi: 10.1186/1471-2105-9-248.

[5] S. Rouskin, M. Zubradt, S. Washietl, M. Kellis, and J.S. Weissman. Genome-wide probing of RNA structure reveals active unfolding of mRNA structures in vivo. *Nature*, 505(7485):701–705, Jan 2014. doi: 10.1038/nature12894.

[6] C. Anthon, H. Tafer, J.H. Havgaard, B. Thomsen, J. Hedegaard, S.E. Seemann, S. Pundhir, S. Kehr, S. Bartschat, M. Nielsen, R.O. Nielsen, M. Fredholm, P.F. Stadler, and J. Gorodkin. Structured RNAs and synteny regions in the pig genome. *BMC Genomics*, 15:459, Jun 2014. doi: 10.1186/1471-2164-15-459.

[7] A.R. Quinlan and I.M. Hall. BEDTools: a flexible suite of utilities for comparing genomic features. *Bioinformatics*, 26(6):841–842, Mar 2010. doi: 10.1093/bioinformatics/btq033.

[8] J Harrow, A Frankish, J M Gonzalez, E Tapanari, M Diekhans, F Kokocinski, B L Aken, D Barrell, A Zadissa, S Searle, I Barnes, A Bignell, V Boychenko, T Hunt, M Kay, G Mukherjee, J Rajan, G Despacio-Reyes, G Saunders, C Steward, R Harte, M Lin, C Howald, A Tanzer, T Derrien, J Chrast, N Walters, S Balasubramanian, B Pei, M Tress, J M Rodriguez, I Ezkurdia, J van Baren, M Brent, D Haussler, M Kellis, A Valencia, A Reymond, M Gerstein, R Guigo, and T J Hubbard. {GENCODE}: The reference human genome annotation for The {ENCODE} Project. *Genome Res*, 22(9):1760–1774, September 2012. doi: 10.1101/gr.135350.111.

[9] S W Burge, J Daub, R Eberhardt, J Tate, L Barquist, E P Nawrocki, S R Eddy, P P Gardner, and A Bateman. Rfam 11.0: 10 years of {RNA} families. *Nucleic Acids Res*, 41(Database issue):D226–32, January 2013. doi: 10.1093/nar/gks1005.

[10] A Kozomara and S Griffiths-Jones. {miRBase}: integrating {microRNA} annotation and deep-sequencing data. *Nucleic Acids Res*, 39(Database issue):D152–7, January 2011. doi: 10.1093/nar/gkq1027.

[11] Laurent Lestrade and Michel J Weber. snoRNA-LBME-db, a comprehensive database of human H/ACA and C/D box snoRNAs. *Nucleic Acids Res*, 34(Database issue):D158—-D162, January 2006. ISSN 1362-4962. doi: 10.1093/nar/gkj002.

[12] H. Hezroni, D. Koppstein, M.G. Schwartz, A. Avrutin, D.P. Bartel, and I. Ulitsky. Principles of long noncoding RNA evolution derived from direct comparison of transcriptomes in 17 species. *Cell Rep*, 11(7): 1110–1122, May 2015. doi: 10.1016/j.celrep.2015.04.023.

[13] M M Hoffman, J Ernst, S P Wilder, A Kundaje, R S Harris, M Libbrecht, B Giardine, P M Ellenbogen, J A Bilmes, E Birney, R C Hardison, I Dunham, M Kellis, and W S Noble. Integrative annotation of chromatin elements from {ENCODE} data. *Nucleic Acids Res*, 41(2):827–841, January 2013. doi: 10.1093/nar/gks1284.

[14] J. Wang, J. Zhuang, S. Iyer, X. Lin, T.W. Whitfield, M.C. Greven, B.G. Pierce, X. Dong, A. Kundaje, Y. Cheng, O.J. Rando, E. Birney, R.M. Myers, W.S. Noble, M. Snyder, and Z. Weng. Sequence features and chromatin structure around the genomic regions bound by 119 human transcription factors. *Genome Res*, 22(9):1798–1812, Sep 2012. doi: 10.1101/gr.139105.112.

[15] Jasmina Ponjavic, Chris P Ponting, and Gerton Lunter. Functionality or transcriptional noise? Evidence for selection within long noncoding RNAs. *Genome Res*, 17(5):556–565, 2007. ISSN 1088-9051. doi: 10.1101/gr.6036807.

[16] G Lunter, C P Ponting, and J Hein. Genome-wide identification of human functional {DNA} using a neutral indel model. *PLoS Comput Biol*, 2(1):e5, January 2006. doi: 10.1371/journal.pcbi.0020005.

[17] A. Hodgkinson, F. Casals, Y. Idaghdour, J.C. Grenier, R.D. Hernandez, and P. Awadalla. Selective constraint, background selection, and mutation accumulation variability within and between human populations. *BMC Genomics*, 14:495, 2013. doi: 10.1186/1471-2164-14-495.

[18] M N Cabili, C Trapnell, L Goff, M Koziol, B Tazon-Vega, A Regev, and J L Rinn. Integrative annotation of human large intergenic noncoding {RNAs} reveals global properties and specific subclasses. *Genes Dev*, 25(18):1915–1927, September 2011. doi: 10.1101/gad.17446611.

[19] D.C. Jones, W.L. Ruzzo, X. Peng, and M.G. Katze. A new approach to bias correction in RNA-seq. *Bioinformatics*, 28(7):921–928, Apr 2012. doi: 10.1093/bioinformatics/bts055.

[20] ENCODE Project Consortium. An integrated encyclopedia of DNA elements in the human genome. *Nature*, 489(7414):57–74, Sep 2012. doi: 10.1038/nature11247.

[21] D. Brawand, M. Soumillon, A. Necsulea, P. Julien, G. Csardi, P. Harrigan, M. Weier, A. Liechti, A. Aximu-Petri, M. Kircher, F.W. Albert, U. Zeller, P. Khaitovich, F. Grutzner, S. Bergmann, R. Nielsen, S. Paabo, and H. Kaessmann. The evolution of gene expression levels in mammalian organs. *Nature*, 478 (7369):343–348, Oct 2011. doi: 10.1038/nature10532.

[22] A. Dobin, C.A. Davis, F. Schlesinger, J. Drenkow, C. Zaleski, S. Jha, P. Batut, M. Chaisson, and T.R. Gingeras. STAR: ultrafast universal RNA-seq aligner. *Bioinformatics*, 29(1):15–21, Jan 2013. doi: 10.1093/bioinformatics/bts635.

[23] C.H. Bang-Berthelsen, L. Pedersen, T. Floyel, P.H. Hagedorn, T. Gylvin, and F. Pociot. Independent component and pathway-based analysis of miRNA-regulated gene expression in a model of type 1 diabetes. *BMC Genomics*, 12:97, 2011. doi: 10.1186/1471-2164-12-97.

[24] W. Rychlik. OLIGO 7 primer analysis software. *Methods Mol Biol*, 402:35–60, 2007. doi: 10.1007/978-1-59745-528-2_2.

[25] A. Untergasser, H. Nijveen, X. Rao, T. Bisseling, R. Geurts, and J.A. Leunissen. Primer3plus, an enhanced web interface to primer3. *Nucleic Acids Res*, 35(Web Server issue):W71–4, Jul 2007. doi: 10.1093/nar/gkm306.

[26] J. Vandesompele, K. De Preter, F. Pattyn, B. Poppe, N. Van Roy, A. De Paepe, and F. Speleman. Accurate normalization of real-time quantitative RT-PCR data by geometric averaging of multiple internal control genes. *Genome Biol*, 3(7):RESEARCH0034, Jun 2002.

[27] N. Hecker, M. Christensen-Dalsgaard, S.E. Seemann, J.H. Havgaard, P.F. Stadler, I.L. Hofacker, H. Nielsen, and J. Gorodkin. Optimizing RNA structures by sequence extensions using RNAcop. *Nucleic Acids Res*, 43(17):8135–8145, Sep 2015. doi: 10.1093/nar/gkv813.

[28] A Untergasser, I Cutcutache, T Koressaar, J Ye, B C Faircloth, M Remm, and S G Rozen. Primer3–new capabilities and interfaces. *Nucleic Acids Res*, 40(15):e115, August 2012. doi: 10.1093/nar/gks596.

[29] J Pan, D Thirumalai, and S A Woodson. Folding of {RNA} involves parallel pathways. *J Mol Biol*, 273 (1):7–13, October 1997. doi: 10.1006/jmbi.1997.1311.

[30] P E Auron, L D Weber, and A Rich. Comparison of transfer ribonucleic acid structures using cobra venom and S1 endonucleases. *Biochemistry*, 21(19):4700–4706, September 1982.

[31] P Gornicki, F Baudin, P Romby, M Wiewiorowski, W Kryzosiak, J P Ebel, C Ehresmann, and B Ehresmann. Use of lead({II}) to probe the structure of large {RNA}'s. Conformation of the 3' terminal domain of E. coli 16S {rRNA} and its involvement in building the {tRNA} binding sites. *J Biomol Struct Dyn*, 6(5):971–984, April 1989. doi: 10.1080/07391102.1989.10506525.

[32] W R Boorstein and E A Craig. Primer extension analysis of {RNA}. *Methods Enzymol*, 180:347–369, 1989.
