# Supplementary material for: The identification and functional annotation of RNA structures conserved in vertebrates
Source: Genome Res. 2017 Aug;27(8):1371–83. doi: 10.1101/gr.208652.116 (PMC5538553; doi:10.1101/gr.208652.116)
Supplement: Supplemental Material [file supp_gr.208652.116_Supplemental_Fig_S1.pdf]

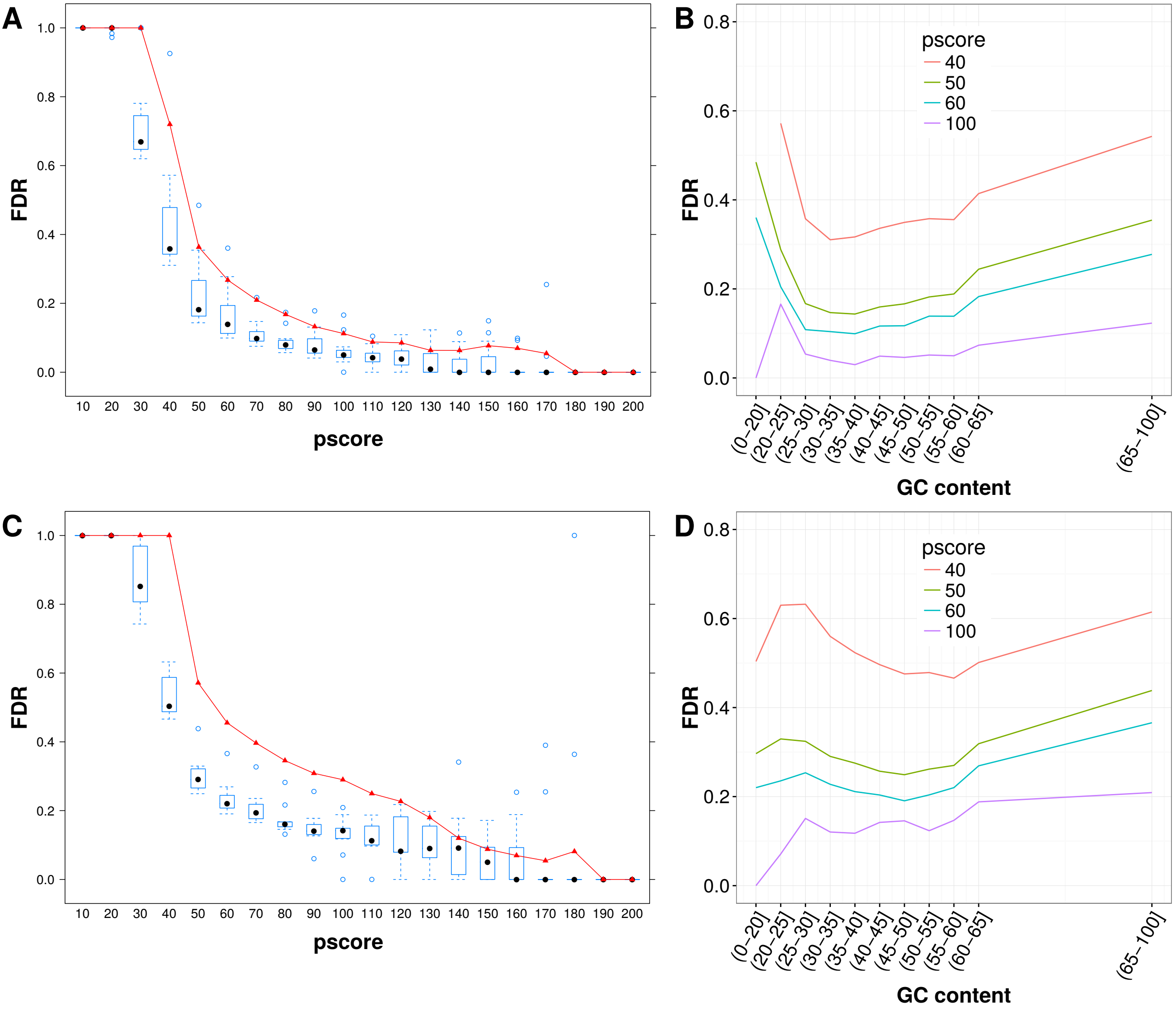

Supplemental Figure S1. False discovery rate (FDR) of CRSs. To assess the prediction performance of CRSs we assigned an *in silico* false discovery rate (FDR) to each CRS by comparing its confidence score (pscore) to `CMfinder` predictions derived from dinucleotide shuffled alignments of similar GC content. The FDRs were calculated for the same GC content range of input multiple alignment (MA) blocks and dinucleotide shuffled MA blocks: (0-20], (20-25], (25-30], (30-35], (35-40], (40-45], (45-50], (50-55], (55-60], (60-65], and (65-100]. As false positives we used `CMfinder` predictions in 17-way MULTIZ alignments that were dinucleotide shuffled by (A,B) `SISSIz` (Gesell *et al.*) and (C,D) `multiperm` (Anandam *et al.*). While the *in silico* mean FDR estimated with `SISSIz` is 15.0±6.6% for CRSs with a GC-content of their input MA block between 20% and 65%, the mean FDR is 14.0±4.9% for CRSs within the same GC-content range measured for their `CMfinder` predicted alignments. The Pearson's correlation coefficient between GC-content of MA blocks (mean=45.6%) and the corresponding CRS alignments (mean=47.1%) is very high with 0.92. (A,C) FDR distribution for different pscore cutoffs. The red triangles are the mean FDRs over all CRSs. The box-and-whisker plots show the FDR distribution for the 11 GC content ranges of the input MA blocks. (B,D) FDR distribution at specific pscore cutoffs for the 11 GC content ranges of the input MA blocks.
