# Supplementary material for: The identification and functional annotation of RNA structures conserved in vertebrates
Source: Genome Res. 2017 Aug;27(8):1371–83. doi: 10.1101/gr.208652.116 (PMC5538553; doi:10.1101/gr.208652.116)
Supplement: Supplemental Material [file supp_gr.208652.116_Supplemental_Fig_S2.pdf]

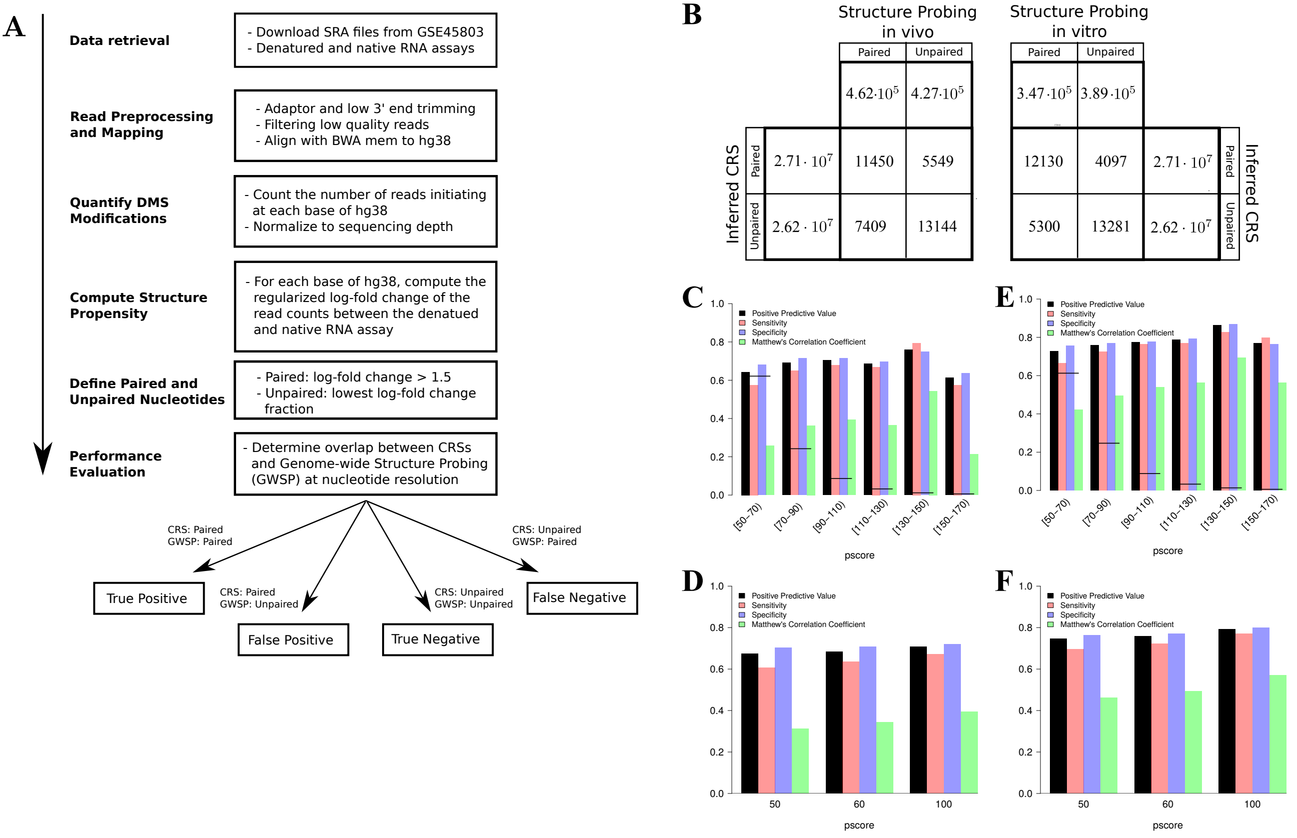

G

Experiment	Fraction	Sensitivity	Specificity	PPV	MCC	Odds-ratio
In vitro	All	0.67	0.74	0.83	0.38	2.22
	Top 20%	0.71	0.75	0.85	0.43	2.56
In vivo	All	0.60	0.67	0.72	0.26	1.67
	Top 20%	0.64	0.68	0.74	0.31	1.88

PPV: Positive Predictive Value

MCC: Mathews Correlation Coefficient

Supplemental Figure S2. Performance of CRS consensus structure using genome-wide structure probing as gold standard. (*A*) The analysis steps used to process data from the DMS based genome-wide structure probing (GWSP). The performance of the CRSs was evaluated at nucleotide resolution by comparing the structure propensity derived from GWSP to the consensus structures of the CRSs. Paired nucleotides were assigned as positives and unpaired nucleotides as negatives. The flow chart should be read from the top and down. (*B*) Contingency table of paired and unpaired nucleotides for the genomic positional overlap of the CRS (pscore$\geq$50) consensus structures and the genome-wide structure probing of the K562 cell line (Rouskin *et al.*). The flanking squares hold the number of positions for which the sets do not overlap and these are thus excluded from the performance assessment. (*C*-*F*) The performance of the CRS consensus structures stratified according to CMfinder score (pscore) (trace *C*,*E*) or based on a pscore cutoff (trace *D*,*F*). The performance was evaluated at nucleotide resolution using the genome-wide structure probing. Horizontal black lines are superimposed on the bar charts to illustrate the fraction of the total overlap that the given pscore stratum constitutes. Traces (*C*,*D*) depict the performance using the *in vivo* condition, while trace (*E*,*F*) depict the *in vitro* condition. There is a general trend of increasing positive predictive values for increasing pscores. (*G*) The performance for all CRSs (pscore$\geq$50).
