# Supplementary material for: The identification and functional annotation of RNA structures conserved in vertebrates
Source: Genome Res. 2017 Aug;27(8):1371–83. doi: 10.1101/gr.208652.116 (PMC5538553; doi:10.1101/gr.208652.116)
Supplement: Supplemental Material [file supp_gr.208652.116_Supplemental_Fig_S3.pdf]

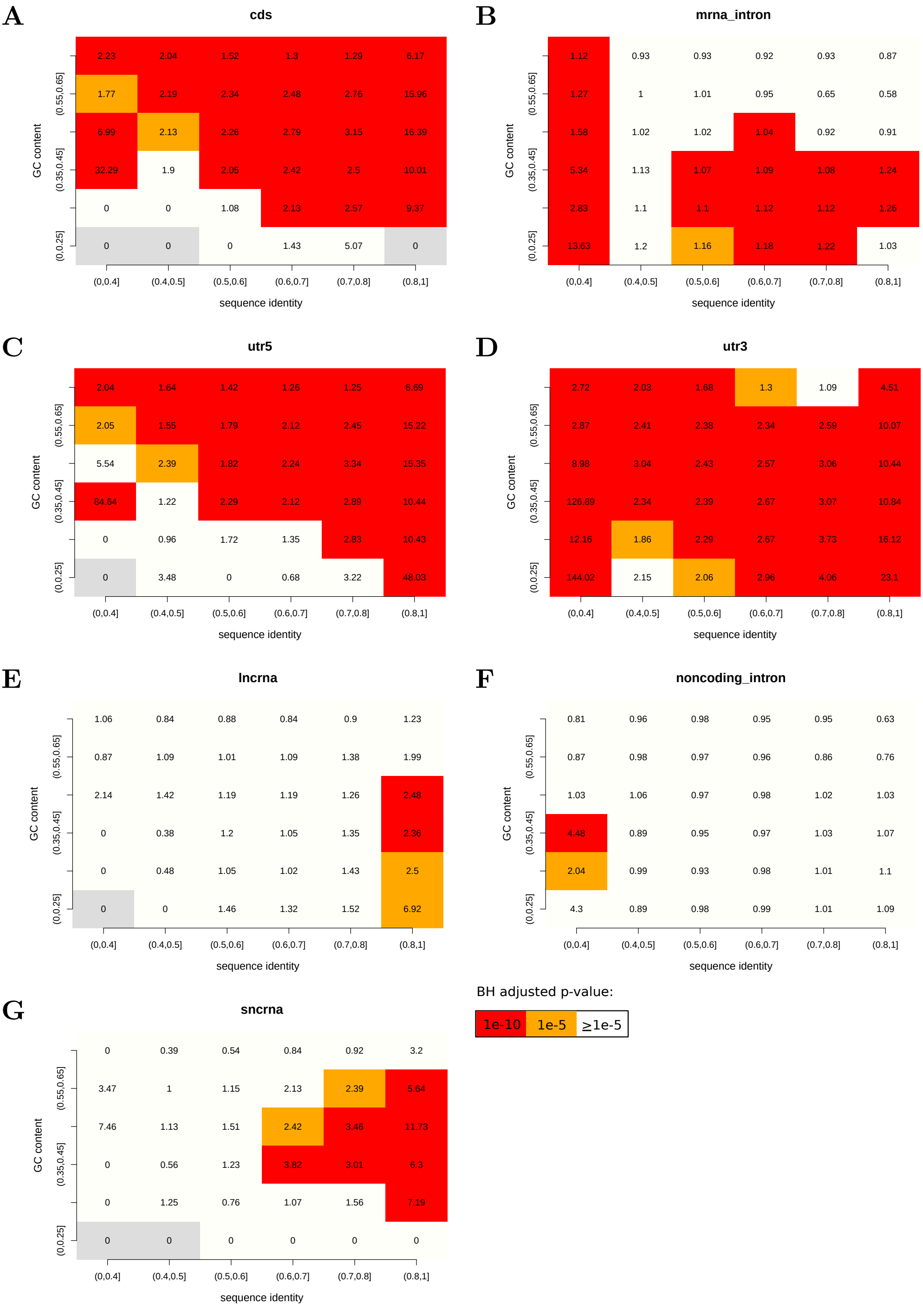

Supplemental Figure S3. Enrichment of CRSs for mRNAs, lncRNAs and small ncRNAs. One-sided Z-test with BH adjusted p-values of CRS enrichment for different biotypes. (*A*) mRNA coding sequence (CDS), (*B*) mRNA intron, (*C*) 5' UTR, (*D*) 3' UTR, (*E*) lncRNA exon, (*F*) lncRNA intron, (*G*) small ncRNA. Biotypes were annotated with GENCODE v25 (Harrow *et al.*) and RNAnnotator pipeline (Anthon *et al.*) for different GC contents and sequence identities of 100bp long windows of concatenated MULTIZ blocks (see Methods). The number in each cell is fold enrichment. The color scheme of BH adjusted p-values: red – $p \leq 10^{-10}$, orange – $p \leq 10^{-5}$, white – $p > 10^{-5}$, gray – too little data.
