# Supplementary material for: The identification and functional annotation of RNA structures conserved in vertebrates
Source: Genome Res. 2017 Aug;27(8):1371–83. doi: 10.1101/gr.208652.116 (PMC5538553; doi:10.1101/gr.208652.116)
Supplement: Supplemental Material [file supp_gr.208652.116_Supplemental_Fig_S4.pdf]

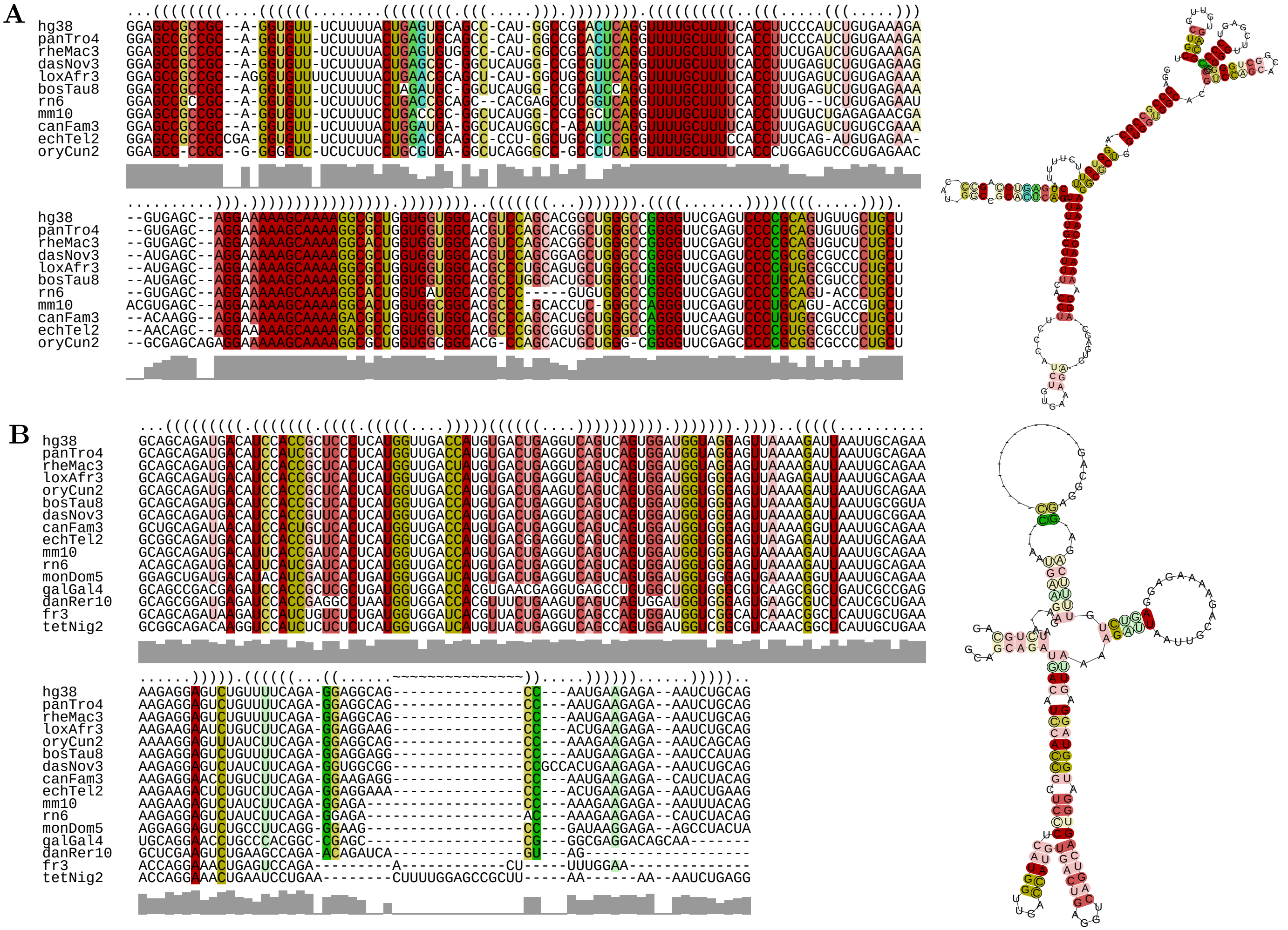

Supplemental Figure S4. Examples of structured domains in lncRNAs. (*A*) tRNA-like structure in the 3' end of NEAT1. `CMfinder` predicts the 3' end of NEAT1 as highly structured. CRS *M0235907* (pscore=110.19, FDR=2.25) includes two stems (stem 4 and 5 counted from 5' end) that perfectly match the cloverleaf structure of the NEAT1 tRNA-like small RNA described in (Sunwoo *et al.*), whereas the other two stems of the cloverleaf structure fold back to the 3' end of NEAT1 (stem 1). In addition, U-rich motif 2 and the encoded short poly(A) tail-like moiety are perfectly complementary (stem 3), and between U-rich motif 1 and 2 a conserved stem-loop is predicted as suggested in (Sunwoo *et al.*) (stem 2). (*B*) Three-way junction of the SRA1 lncRNA. `CMfinder` predicts the CRS *M2215683* (pscore=66.87, FDR=11.72) that overlaps the three-way junction branching helices H15, H16 and H17 in the domain III of the SRA1 lncRNA (and most 3' coding exon of SRA1) as described in (Novikova *et al.*).
