# Supplementary material for: The identification and functional annotation of RNA structures conserved in vertebrates
Source: Genome Res. 2017 Aug;27(8):1371–83. doi: 10.1101/gr.208652.116 (PMC5538553; doi:10.1101/gr.208652.116)
Supplement: Supplemental Material [file supp_gr.208652.116_Supplemental_Fig_S5.pdf]

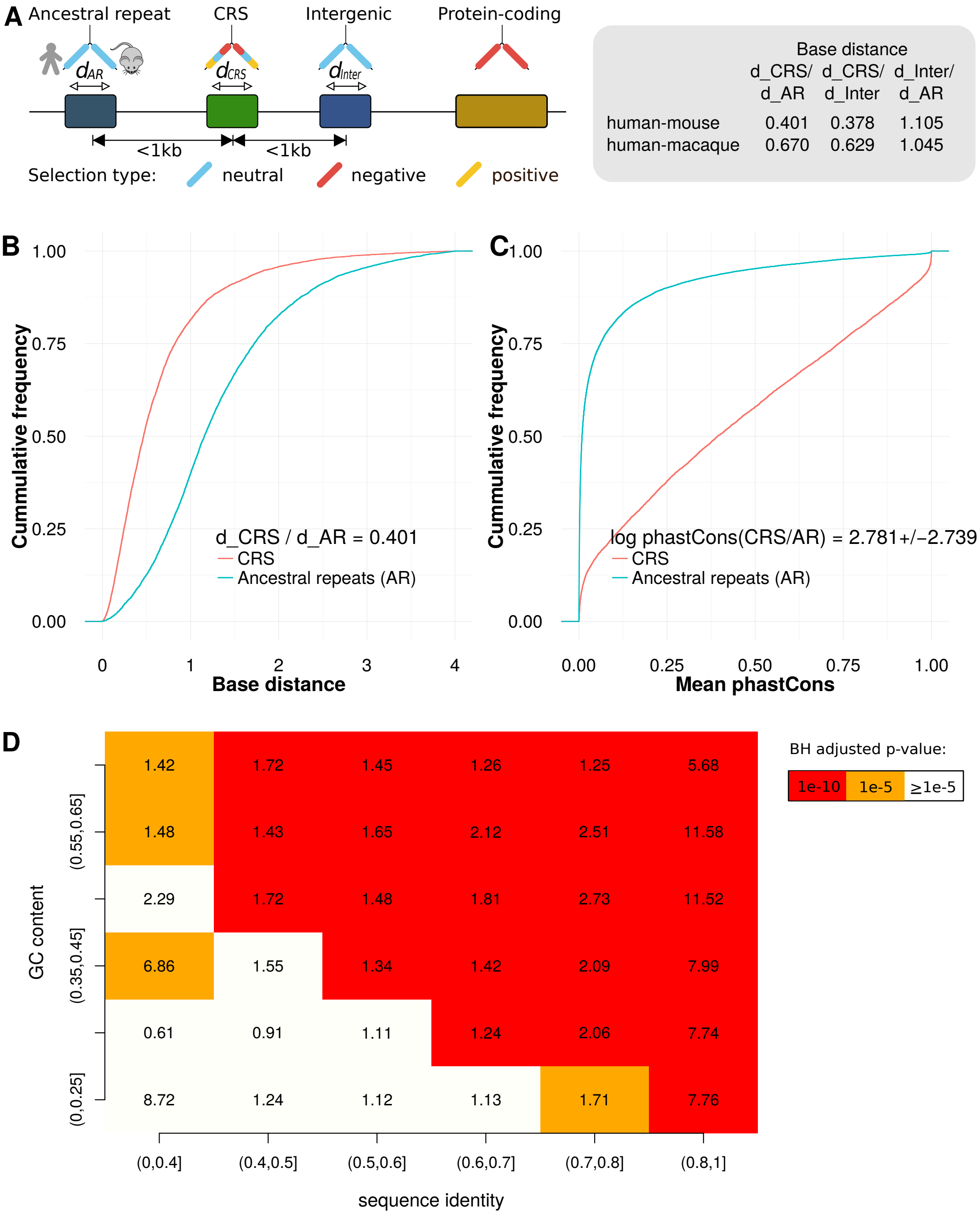

Supplemental Figure S5. Evolutionary selection within CRSs. (*A*) The cartoon illustrates a model where ancestral repeats and intergenic regions are under neutral selection, while protein-coding sequences are under negative selection. Distances are calculated between human and mouse as well as human and rhesus macaque for CRSs (d_{CRS}), local ancestral repeats (d_{AR}) and local intergenic sequences (d_{Inter}). The both latter are independent sets of putative neutral evolving sequences. We hypothesize that if the ratio is 1 then selection has not distinguished substitutions between the numerator and the denominator. If the ratio is significantly less than 1, then this is an indication that either purifying selection on substitutions has been more prevalent in the numerator, or that underlying mutation rates are lower in the numerator. (*B*) The actual cumulative frequency of base distance d between human and mouse in CRSs (d_{CRS}) and local ancestral repeats (d_{AR}) showed lower base distances in CRSs. (*C*) Cumulative frequency of mean phastCons scores of CRSs and ancestral repeats. Mean phastCons are the average of phastCons scores in the respective region (CRS, AR). The *log phastCons* are mean and standard deviation of phastCons-based ratios. The selection analysis based on phastCons scores supports that nucleotide substitution is suppressed in CRSs which is a signature of purifying selection for the majority of measured CRSs. (*D*) Enrichment of CRSs for segments under indel-purifying selection (Lunter *et al.* 2006). Colored squares flag GC:SI bins having fewer indel mutations near CRSs than expected by chance (one-sided Z-test BH corrected). We considering only CRSs >10kb from the closest GENCODE annotated mRNA.
