# Supplementary figures and images for: The identification and functional annotation of RNA structures conserved in vertebrates
Source: Genome Res. 2017 Aug;27(8):1371–83. doi: 10.1101/gr.208652.116 (PMC5538553; doi:10.1101/gr.208652.116)

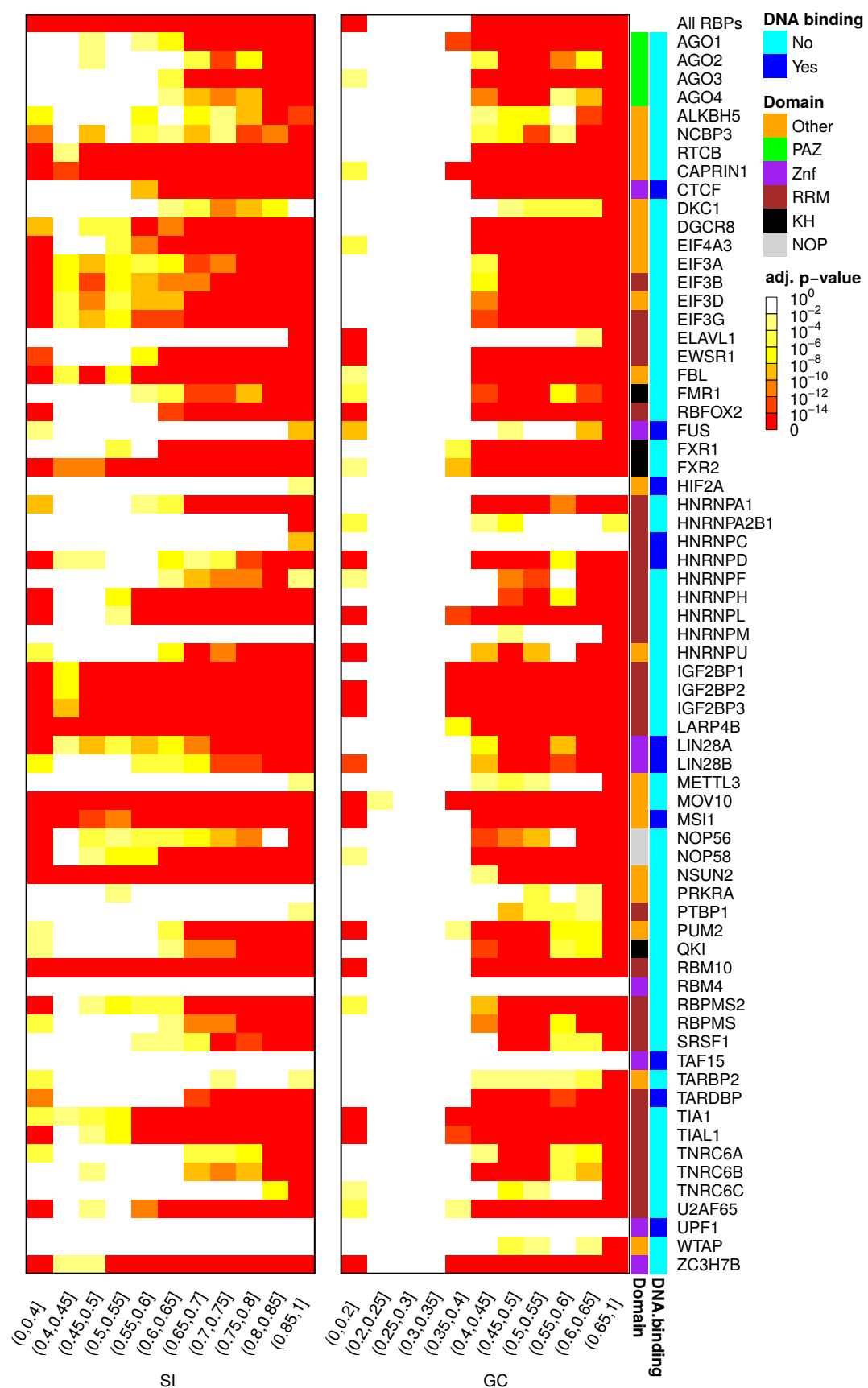

Supplement: Supplemental Material [file supp_gr.208652.116_Supplemental_Fig_S6.pdf]
