# Supplementary material for: The identification and functional annotation of RNA structures conserved in vertebrates
Source: Genome Res. 2017 Aug;27(8):1371–83. doi: 10.1101/gr.208652.116 (PMC5538553; doi:10.1101/gr.208652.116)
Supplement: Supplemental Material [file supp_gr.208652.116_Supplemental_Fig_S7.pdf]

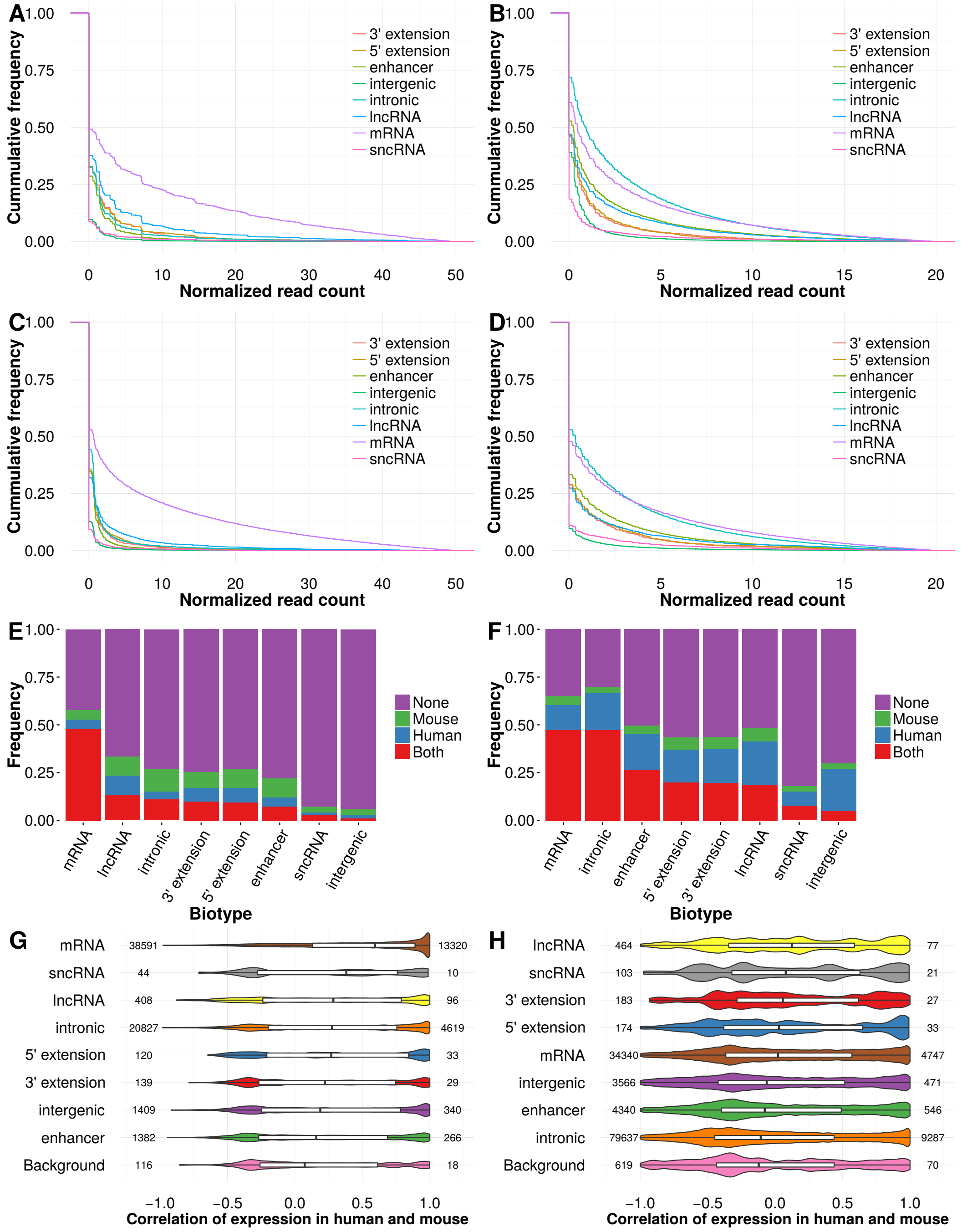

Supplemental Figure S7. Expression conservation in human and mouse. To characterize the expression of CRSs of different biotypes in different tissues we analyzed (*A*,*C*,*E*,*G*) poly(A)-selected RNA and (*B*,*D*,*F*,*H*) total RNA. Input were 433,327 CRSs with the same biotype in human and mouse out of the 543,390 CRSs conserved in human and mouse. (*A*-*D*) Cumulative frequency of normalized read counts (CPM/RLE) in (*A*,*B*) human and (*C*,*D*) mouse. Read counts were from 201bp long windows around CRSs. For each CRS the maximum normalized read count in all tissues was considered. (*E*,*F*) The fraction of CRSs that was detected as expressed in human or mouse or both. CRSs with empirical $P < 0.01$ of expression in ≥ 1 tissue were assigned an "expressed" state. (*G*,*H*) The similarity of expression levels of CRSs over all tissues was examined between human and mouse. First, we normalized the raw read counts over all human and mouse tissues (CPM and RLE normalization with edgeR). Second, we assigned the mean value of normalized read counts from the replicates to each tissue. Third, we calculated the Pearson correlation coefficient between the tissue vectors for human and mouse for all CRSs. The figures show the correlation coefficients for the different biotypes with CRSs filtered for expression in at least 2 tissues in both human and mouse. The total number of CRSs is listed on the left side of boxplots and the number of CRSs with Pearson correlation ≥0.8 on the right side. The "Background" are 201bp long windows sampled from the input MA blocks with human-mouse conservation and without overlap to the other biotypes.
