# Supplementary material for: The identification and functional annotation of RNA structures conserved in vertebrates
Source: Genome Res. 2017 Aug;27(8):1371–83. doi: 10.1101/gr.208652.116 (PMC5538553; doi:10.1101/gr.208652.116)
Supplement: Supplemental Material [file supp_gr.208652.116_Supplemental_Fig_S8.pdf]

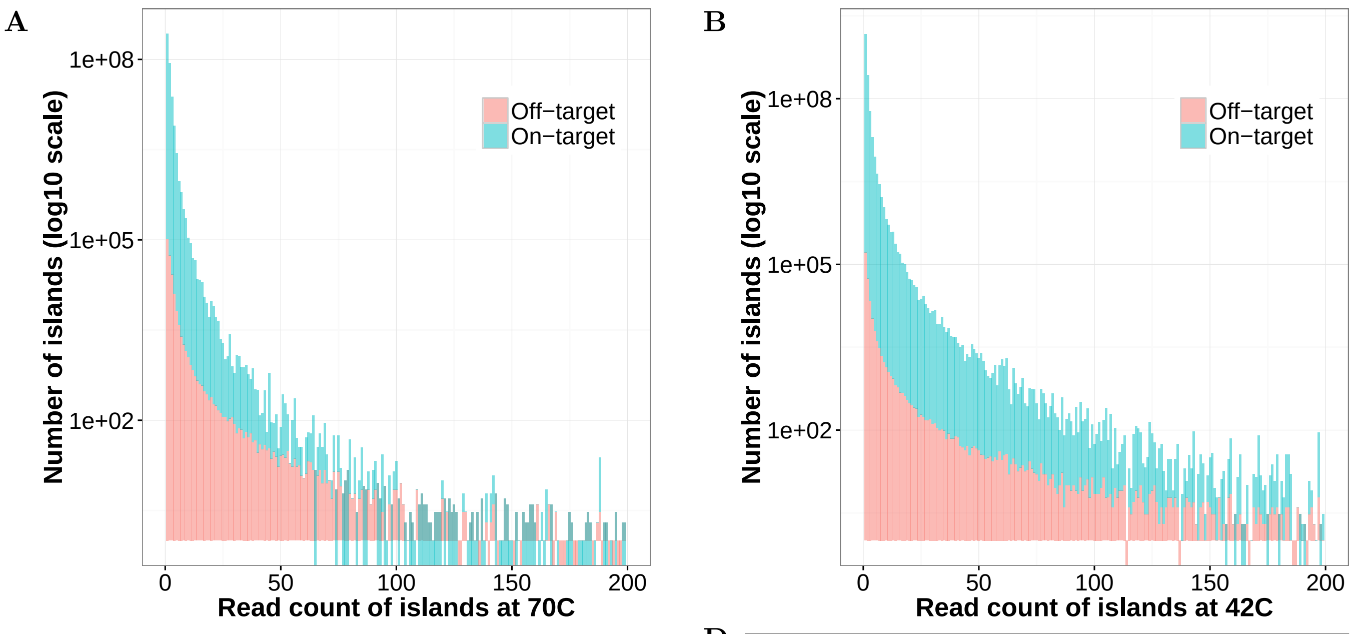

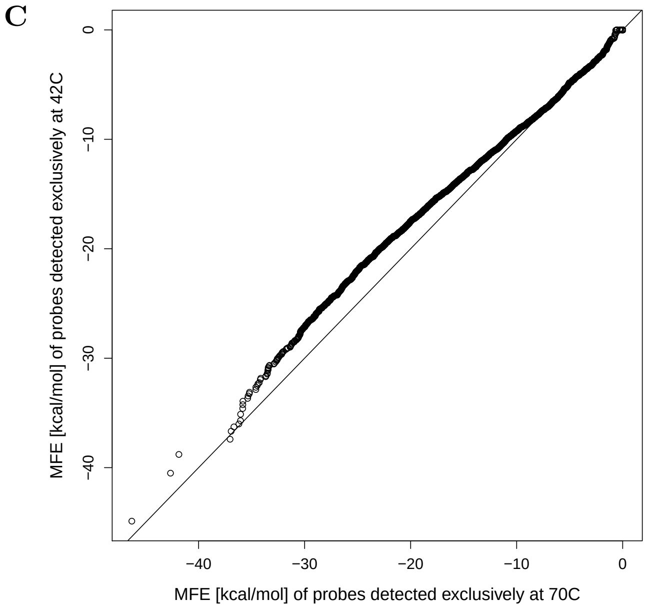

D

P-value	Read count	Number read islands	Number CRS regions
**70°C**			
0.25	4	3,228	2,593
0.1	6	2,190	1,804
0.05	9	1,518	1,288
0.01	28	557	458
**42°C**			
0.25	3	18,783	12,239
0.1	6	12,687	8,326
0.05	10	9,307	6,170
0.01	34	4,100	2,727

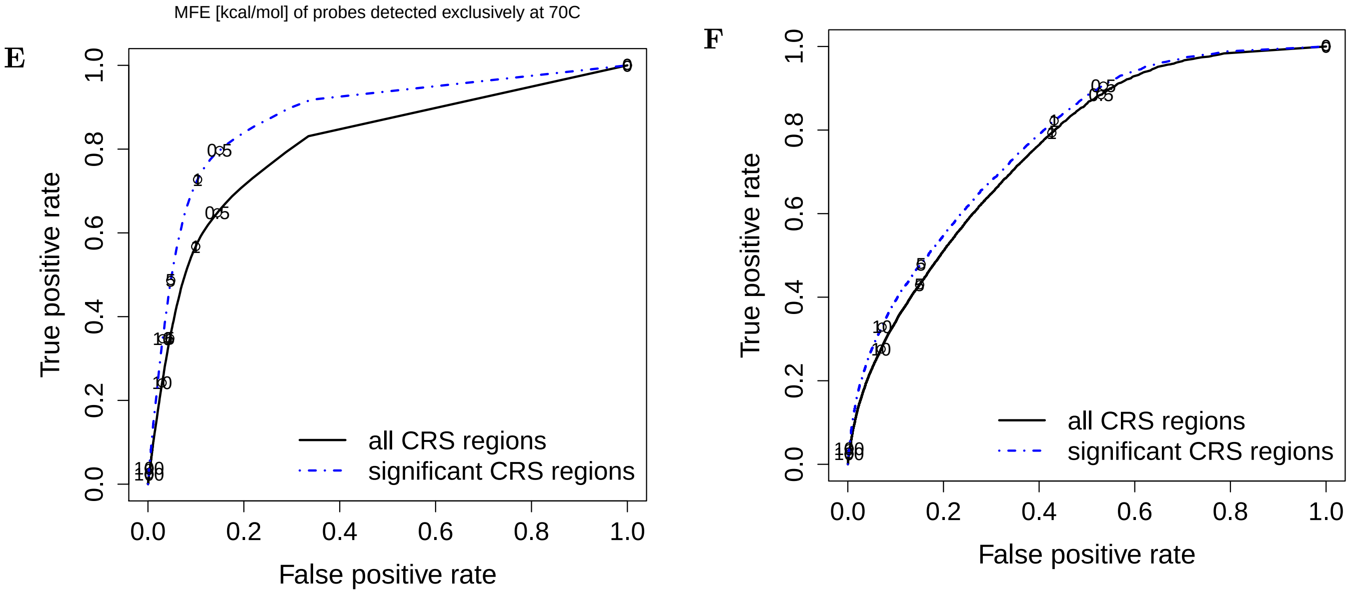

Supplemental Figure S8. CaptureSeq to enrich CRS expression signals. We aimed to detect novel transcripts by extending the project pipeline to a large scale customized CaptureSeq followed by qRT-PCR. 250k RNA probes were designed for targeting 77,320 CRS regions conserved in human and mouse (2 × 125k probes to target both strands of CRSs), of which most were intergenic. 150 nts long transcript fragments were annealed to the capture array why we chose loci (overlapping reads) with maximal distance of 150 bp to a probe as on-targets. The distribution of read counts for CaptureSeq at both (*A*) 70°C and (*B*) 42°C was significantly larger ($P = 0$, one-sided Mann-Whitney U-test) for on-target read islands (overlap an RNA probe) than for off-target read islands (do not overlap an RNA probe). (*C*) Since, we in particular were targeting structured RNA, in contrast to for example more standardized strategies such as less structured coding sequence, and the RNA therefore can have higher melting temperatures (more structured and low folding energy), we experimented with two melting temperatures 42 °C and 70 °C in our settings. The average minimum free energy (MFE) was lower for probes only hybridized at 70°C than for probes only hybridized at 42°C. We found that the highly thermodynamically stable RNAs were bound only at 70 °C, but at the cost of a drop in the detection rate compared to the standard protocol at 42 °C. Thus, most candidates were detectable at 42 °C, but were complemented by the usage of higher annealing temperature for highly thermodynamically stable RNAs. (*D*) We calculated an empirical P-values for different read counts of read islands. The number of RNA probe overlapping read islands and corresponding CRS regions is listed for the different P-values. We chose a P-value P<0.1 to define significantly expressed CRS regions. (*E*,*F*) The ROC graphs treat the captured read island of the CRS CaptureSeq as true positives and evaluate the expression in brain of two selected publicly available RNA-seq data: (*E*) PolyA-selected RNA from Human Body Map 2.0 (1 total brain library) and (*F*) ENCODE phase 3 total RNA (5 brain libraries). The numbers along the curves are the different read counts in CPM/RLE. The black solid curves represent all probe overlapping CRS regions and the blue dashed curves CRS regions with significant read islands (P<0.1).
