# Supplementary material for: The identification and functional annotation of RNA structures conserved in vertebrates
Source: Genome Res. 2017 Aug;27(8):1371–83. doi: 10.1101/gr.208652.116 (PMC5538553; doi:10.1101/gr.208652.116)
Supplement: Supplemental Material [file supp_gr.208652.116_Supplemental_Fig_S9.pdf]

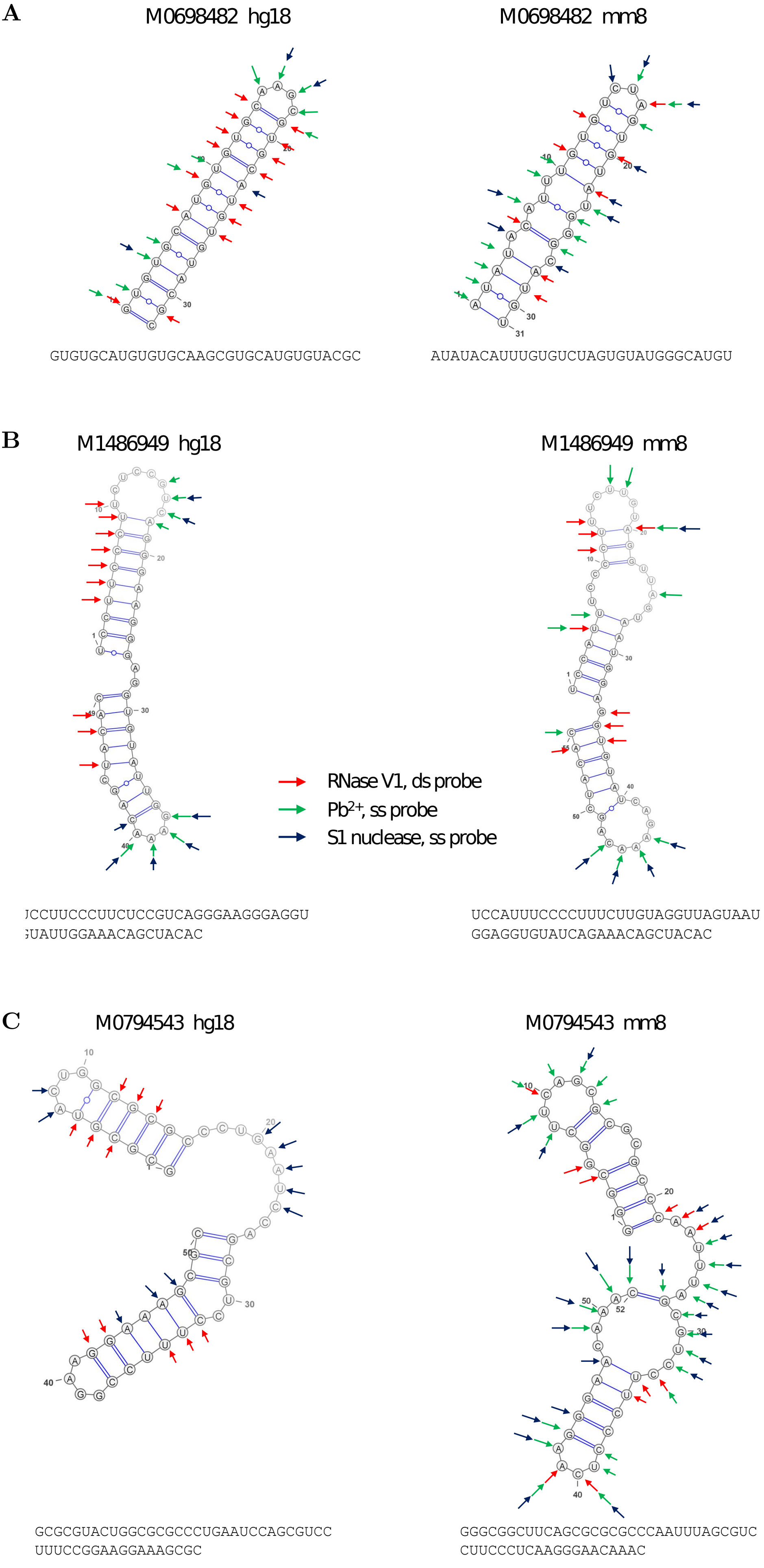

Supplemental Figure S9. RNA structure probing. RNA structure probing in human (hg18) and mouse (mm8) for the CRSs **(a)** *M0698482*, **(b)** *M1486949*, and **(c)** *M0794543*. Red arrows mark nucleotides identified to be basepaired in the probing experiment (ds), and green and blue arrows mark single-stranded nucleotides (ss).
