# Supplementary material for: The identification and functional annotation of RNA structures conserved in vertebrates
Source: Genome Res. 2017 Aug;27(8):1371–83. doi: 10.1101/gr.208652.116 (PMC5538553; doi:10.1101/gr.208652.116)
Supplement: Supplemental Material [file supp_gr.208652.116_Supplemental_Fig_S10.pdf]

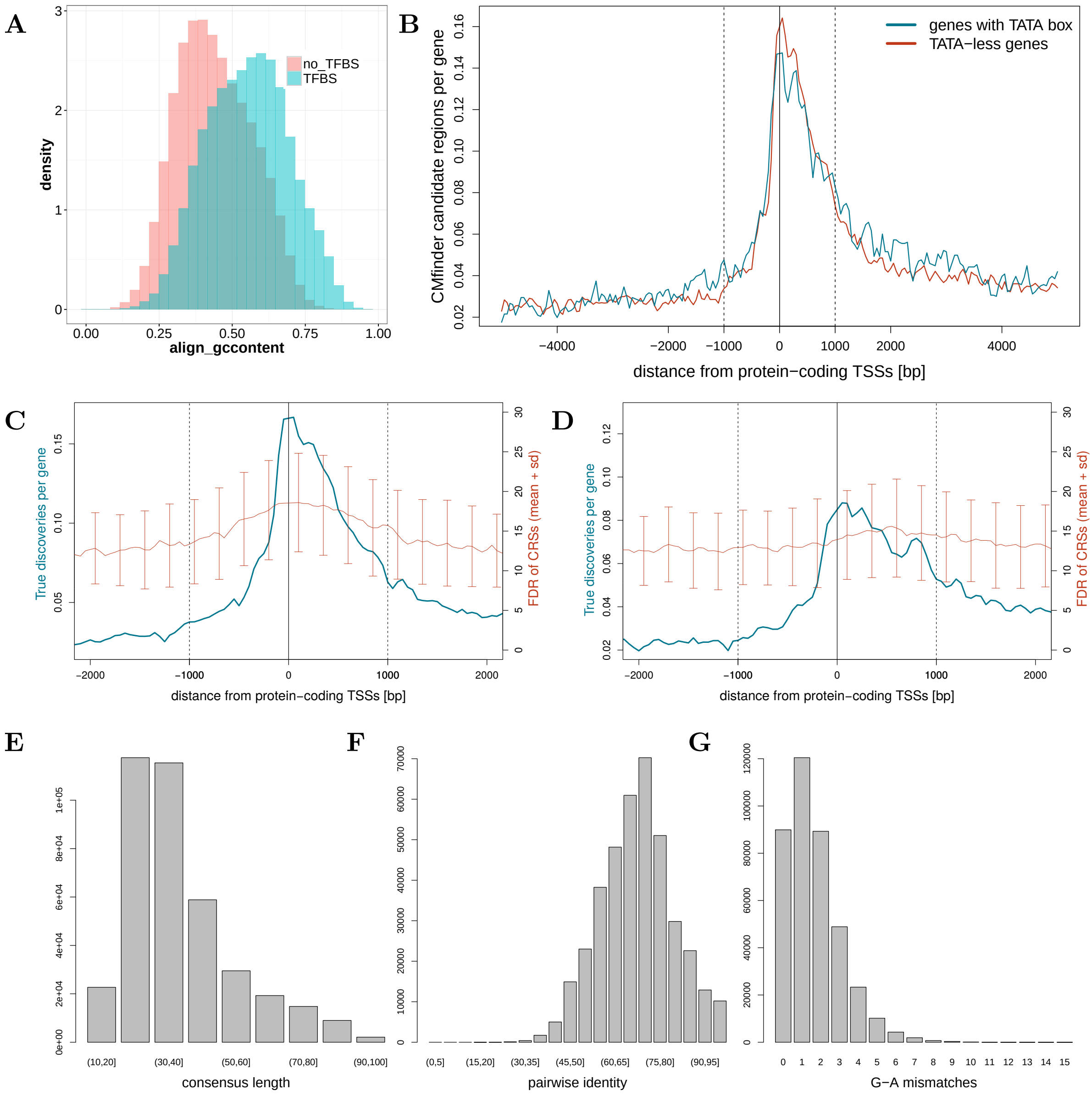

H

	all	TFBS co-localized		
		all	upstream TSS	downstream TSS
CRS number	778,701	152,082	10,124	10,060
simple hairpin	393,184	65,922	4,447	4,471
median GC content [%]	46.1	51.7	56.6	56.5
median compensatory bp changes	6	5	6	6
median SI [%]	60.3	65.6	65.0	64.9
overlapped microRNAs	1,188	288	16	21
median length	55.50	61.6	59.75	59.70
median SI of seq.+rev.compl. [%]	33.5	32.9		
median length of longest palindrome	7	7	8	8

Supplemental Figure S10. Impact of genomic features around TFBSs and TSSs on CRS prediction. A large fraction of CRSs is located at TF binding sites and close to TSSs of annotated genes. (*A*) The GC content is significantly larger for CRSs that overlap TF binding sites compared to CRSs that do not overlap TF binding sites (mean of 53% compared to 44%). (*B*) Genes with TATA boxes in their promoter have, in general, larger directionality than TATA-less genes, however, our study showed similar frequencies of CRSs around TSSs for both types. Step size =50 bp. Selected were 1,765 genes with TATA boxes and 5,154 TATA-less genes (Yang *et al.*) (>10kb distance between studied genes from GENCODE v12). (*C,D*) Genes with CpG islands in their promoters do not have crisply defined TSSs, so candidates "upstream" of annotated TSSs may really be downstream. We looked separately at 7,013 genes with CpG islands and 7,473 genes without CpG islands to see if there is a difference. (*C*) CRS regions that overlapped CpG islands. (*D*) CRS regions that did not overlap CpG islands. The blue curve counts the true discoveries of CRS regions which is equal to (1 - FDR) of overlapped CRSs, and the red bars show standard deviations of CRS's FDR. Despite of more CRS regions around TSSs with CpG islands, the CRS frequency around TSSs without CpG islands is similar with even lower FDRs. (*E,F,G*) Inverted repeats of a few 10's of bases in length are quite common in the genome for various reasons, *e.g.* dimeric TFBSs, transposons, some mutational processes. We checked for the possibility that CRSs are just inverted repeats instead of functional RNA structures. (*E*) We identified 389,417 hairpin structures of length ≤100 bp. (*F,G*) Inverted repeats can have an intervening length of zero (palindrom) to undefined, so we removed the hairpin loop of the predicted consensus structure from the consensus sequence before aligning the sequence with its reverse complement (global alignment: match=+2, mismatch=-1, gap=-2). Potential inverted repeats have high pairwise SI and a low number of G-A mismatches (G-A mismatches might result from G-U wobble basepairs in the hairpin structure). We defined inverted repeats as sequences with SI>90% and G-A mismatches<2 to their reverse complement. Using this definition 23,047 CRSs were classified as inverted repeats, which is, however, only 3.0% of all CRSs. (*H*) Palindromic sequences have previously been found in many recognition sequences of TFs, why we checked the potential for palindromicity of all CRSs, CRSs co-localized to TFBSs and CRSs co-localized to TFBSs and adjacent to TSSs. We ran blastn (-W 5) between the human CRS sequence and its reverse complement. "SI of seq.+rev.compl." is the pairwise SI between sequence and its reverse complement. Even though CRSs were co-localized to TFBSs, compared to all CRSs they comprise a lower rate of simple hairpins and a lower median SI to their reverse complement, why we conclude that TFBS co-localized CRSs were not biased through palindromes. Only the overlap to microRNAs (mirBase v18) significantly differed for CRSs co-localized to TFBSs compared to all CRSs (Z-test p=0.0007). Upstream TSSs: -3kb to -50bp from TSSs (GENCODE v12). Downstream TSSs: +50bp to +3kb from TSSs.
