# Supplementary material for: The identification and functional annotation of RNA structures conserved in vertebrates
Source: Genome Res. 2017 Aug;27(8):1371–83. doi: 10.1101/gr.208652.116 (PMC5538553; doi:10.1101/gr.208652.116)
Supplement: Supplemental Material [file supp_gr.208652.116_Supplemental_Fig_S11.pdf]

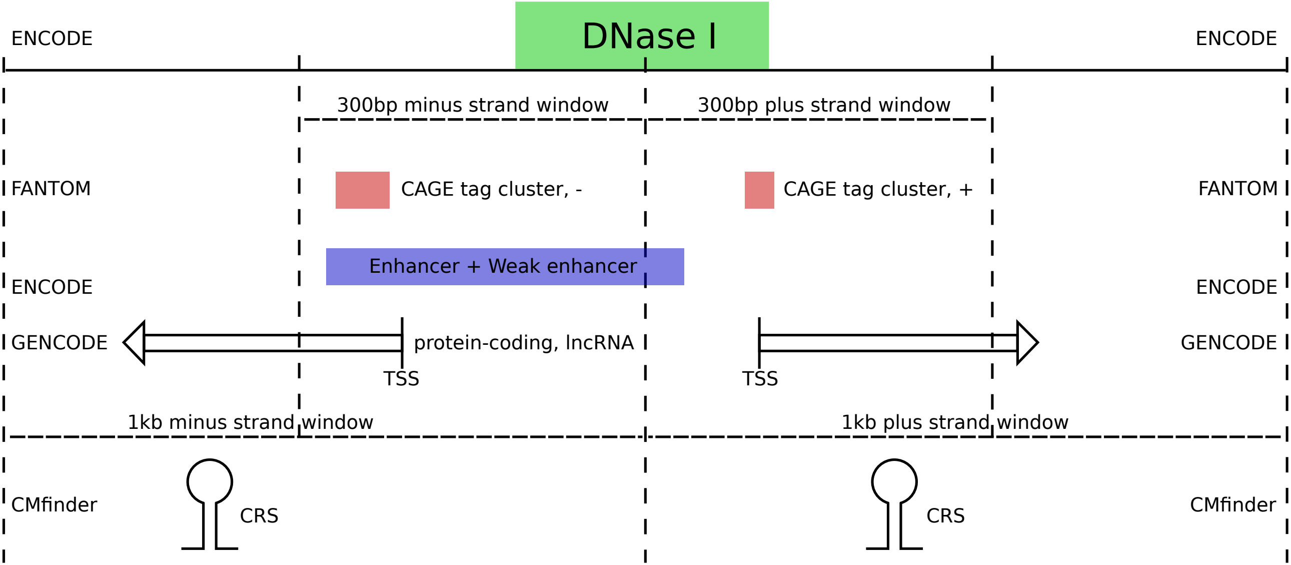


**Supplemental Figure S11.** Schematic illustration of the characterization of structured regulatory regions. We used the following data to define regulatory regions: DNase I Hypersensitivity Peak Clusters from ENCODE (95 cell types), CAGE expression of robust (expression>10TPM) peaks (maximal length of 200bp) from FANTOM5 Phase 2.0, ENCODE chromatin segmentation states and GENCODE v25 gene/TSS annotation of mRNAs and lncRNAs. For the detailed definition of regulatory regions in this study see the Methods section "Definition of gene regulatory regions" and Supplemental Methods.
