# Supplementary material for: The identification and functional annotation of RNA structures conserved in vertebrates
Source: Genome Res. 2017 Aug;27(8):1371–83. doi: 10.1101/gr.208652.116 (PMC5538553; doi:10.1101/gr.208652.116)
Supplement: Supplemental Material [file supp_gr.208652.116_Supplemental_Fig_S12.pdf]

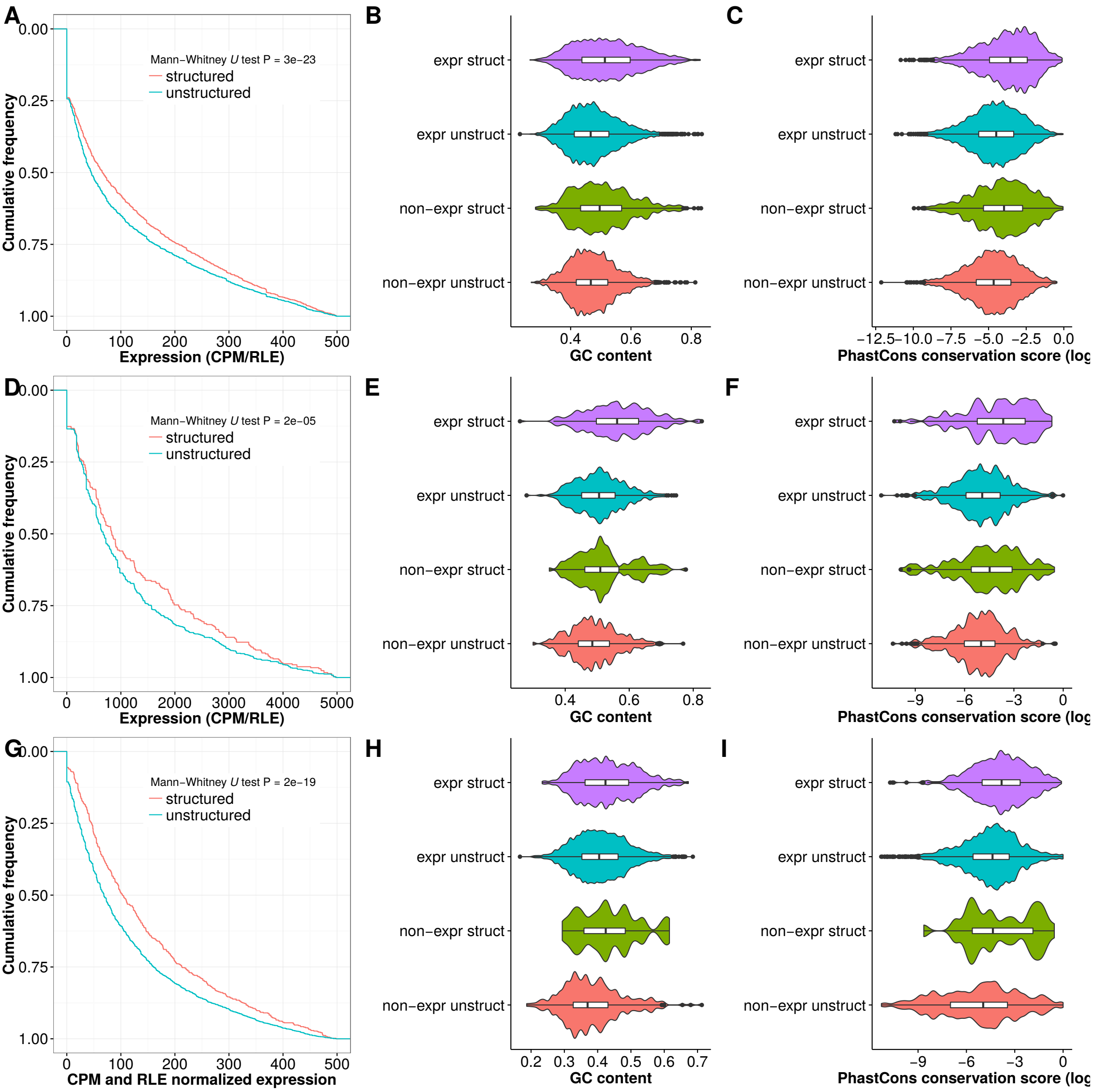

Supplemental Figure S12. Expression of structured versus unstructured regulatory regions. (*A-C*) Enhancer regions. (*D-F*) Loci upstream of mRNA and lncRNA TSSs. (*G-I*) Loci downstream of 3' end of mRNAs and lncRNAs. All considered regulatory regions were supported by transcriptional boundaries (CAGE or poly(A) sites). (*A*,*D*,*G*) Maximal expression level of all considered tissues of structured (overlapping CRS regions) versus unstructured (non overlapping) regulatory regions as cumulative frequency. All three categories were significantly higher expressed if the regulatory region overlapped a CRS region (Mann-Whitney *U*-test). (*A*,*D*) Total RNA-seq (ENCODE phase 3; 19 tissues; 2 replicates) as counts per million (CPM) and relative log expression (RLE) normalized over all tissues. (*G*) Poly(A)-selected RNA-seq (Human Body Map 2.0; 1 tissues; no replicates). However, we also observed a relationship between (*B*,*E*,*H*) GC content, (*C*,*F*,*I*) PhastCons (from 100-species MULTIZ alignments) and CRS regions and expression. Expressed (expr) regulatory regions were defined by normalized read count (CPM/RPM) $\geq$1 in at least one tissue of (*B*,*C*,*E*,*F*) total RNA (ENCODE phase 3; both replicates) or (*H*, *I*) poly(A)-selected RNA (Human Body Map 2.0). Nevertheless, after correcting for GC content and SI we still observed significantly higher expression levels in a large number of tissues for enhancers and loci downstream of 3' ends of mRNAs and lncRNAs (alternative poly(A) sites), see Supplemental Table S5.
