# Supplementary material for: The identification and functional annotation of RNA structures conserved in vertebrates
Source: Genome Res. 2017 Aug;27(8):1371–83. doi: 10.1101/gr.208652.116 (PMC5538553; doi:10.1101/gr.208652.116)
Supplement: Supplemental Material [file supp_gr.208652.116_Supplemental_Fig_S13.pdf]

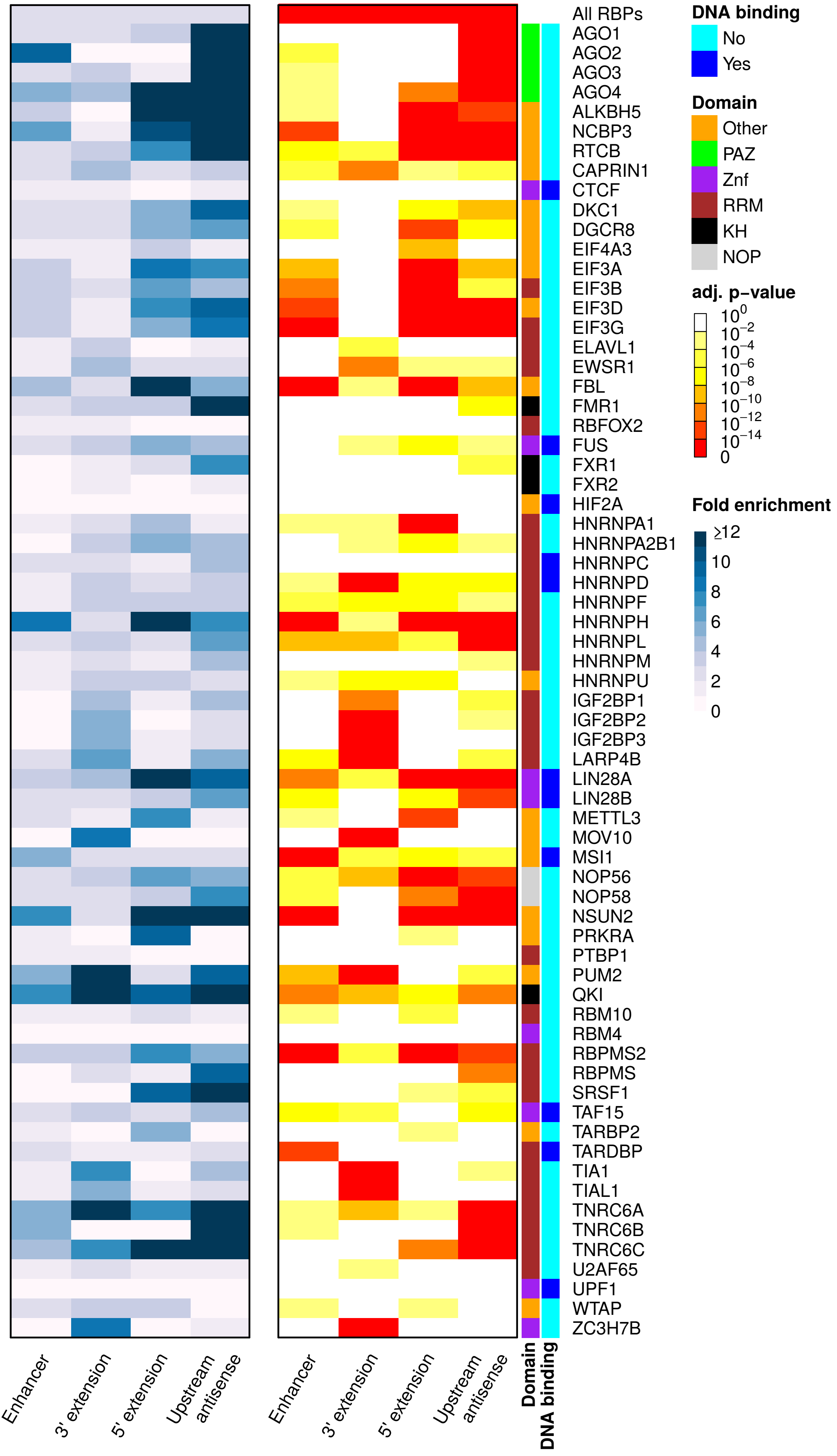


**Supplemental Figure S13.** Enrichment of CRSs localized in regulatory regions for RBP binding sites. The heatmaps show the fold enrichment (left) and the corresponding BH adjusted *p*-values (right) for the enrichment of CRSs located in enhancers, 3' extensions, 5' extensions and upstream antisense of mRNA/lncRNA TSSs for RBP binding sites. The considered regulatory regions were defined by experimentally determined but unannotated transcript boundaries (CAGE TSSs and poly(A) sites).
