# Supplementary material for: The identification and functional annotation of RNA structures conserved in vertebrates
Source: Genome Res. 2017 Aug;27(8):1371–83. doi: 10.1101/gr.208652.116 (PMC5538553; doi:10.1101/gr.208652.116)
Supplement: Supplemental Material [file supp_gr.208652.116_Supplemental_Table_S1.pdf]

Supplemental Table S1. Noncoding RNA annotation in human. CRS regions were intersected with noncoding RNA classes and RNA structure predictions from previous screens in vertebrate genomes. Small RNA paralogues and RNA structure predictions in the input were defined by overlapping at least one input MULTIZ block with ≥50% of their length. Long ncRNAs in the input had to overlap at least one input MULTIZ block with ≥10% of their length because we can not ensure that the majority of the lncRNA length is covered by one MULTIZ block. An overlap of ncRNA paralogues and CRS regions was counted if either ≥50% of the length of the CRS region or ≥50% of the length of the ncRNA paralogue is covered. Only repeat-free sequences were considered (RepeatMasker v4.0.5). The reading direction was ignored. The fold enrichment of CRS regions for the various ncRNA classes compares the fraction of CRS overlapped ncRNAs (or RNA structure predictions) to the expected number of overlaps calculated from the fraction of the input (17-species MULTIZ alignments anchored by hg18) covered by the ncRNA class (measured in bp). In addition, 8 CRS regions co-localized with four 5.8S rRNA pseudogenes out of 19 in the input.

ncRNA class	Reference PMID	Nr. of ncRNA paralogues input	Nr. of ncRNA paralogues overlapping CRSs	Nr. of CRS regions overlapping ncRNA	fold enrichment
small RNAs					
microRNA	24917120	979	269	265	8.84
snRNA	24917120	71	23	24	7.76
tRNA	24917120	116	92	78	21.97
snoRNA	24917120	618	201	208	8.13
long ncRNA exons					
lincRNA exon	22955987	14914	1625	2097	1.60
antisense exon	22955987	12840	1923	2543	2.15
pseudogene exon	22955987	11229	3050	4169	2.34
PLAR exon	25959816	33525	4522	6037	1.81
RNA structure predictions					
EvoFam ECS	21994249	2455	588	583	13.10
RNAz v2.1	19908359	458132	53977	54431	2.87
RNAz & SISSIz	23847102	943524	99082	102799	2.37
