# Supplementary material for: The identification and functional annotation of RNA structures conserved in vertebrates
Source: Genome Res. 2017 Aug;27(8):1371–83. doi: 10.1101/gr.208652.116 (PMC5538553; doi:10.1101/gr.208652.116)
Supplement: Supplemental Material [file supp_gr.208652.116_Supplemental_Table_S2.pdf]

**Supplemental Table S2.** Repeat masked CRSs. 167,415 CRSs overlap repeats from RepeatMasker v4.0.5 (165,966 CRSs) and tandem repeats of length <2kb from TandemRepeatFinder v4.0.4 (3,037 CRSs ). The fold enrichment of CRSs for the various repeat families compares the fraction of CRS overlapped repeats to the expected number of overlaps calculated from the fraction of the input (17-species MULTIZ alignments anchored by hg18) covered by the repeat family (measured in bp).

| Repeat family | Number CRSs | fold enrichment |
|---|---:|---:|
| SINE/MIR | 33,832 | 0.76 |
| LINE/L2 | 23,322 | 0.48 |
| Simple_repeat | 22,611 | 0.96 |
| LINE/L1 | 21,042 | 0.45 |
| DNA/hAT-Charlie | 14,225 | 0.79 |
| LTR/ERVL-MaLR | 10,749 | 0.61 |
| LTR/ERVL | 7,619 | 0.64 |
| LINE/CR1 | 5,365 | 0.77 |
| DNA/TcMar-Tigger | 4,787 | 0.89 |
| Low_complexity | 4,113 | 0.53 |
| DNA/hAT-Tip100 | 3,526 | 0.83 |
| LTR/ERV1 | 1,908 | 0.63 |
| LTR/Gypsy | 1,574 | 0.83 |
| SINE/Alu | 1,572 | 0.28 |
| other | 11,170 | |
