# Supplementary material for: The identification and functional annotation of RNA structures conserved in vertebrates
Source: Genome Res. 2017 Aug;27(8):1371–83. doi: 10.1101/gr.208652.116 (PMC5538553; doi:10.1101/gr.208652.116)
Supplement: Supplemental Material [file supp_gr.208652.116_Supplemental_Table_S3.pdf]

Supplemental Table S3. qRT-PCR candidates. 23 CRS regions were detected by the CaptureSeq experiment ($P < 0.1$) and were further tested by qRT-PCR in human and mouse. Pscore and SI are from the CRSs with the maximal values in the CRS region. Strand ".“ means that overlapping CRSs were predicted on both strands. An *extended UTR* was annotated by GENCODE v25 but was missing in previous GENCODE versions. An CRS region *close to UTR* was located adjacent to an annotated mRNA.

CRS region (hg38)								read island		annotation	
CRS region	chr	start	end	pscore	SI [%]	species	strand	strand	P-value	location	gene name
C2713000	chr20	63403707	63403738	70.34	56.0	36	+	-	0.0090	extended 3'-UTR	KCNQ2
C1785511	chr15	82846711	82846804	77.75	52.64	42	-	-	0.0299	close to 3'-UTR	HOMER2
C0927120	chr12	13557767	13557842	83.99	65.63	56	+	-	0.0111	extended 3'-UTR	GRIN2B
C2213410	chr17	81389084	81389133	75.31	54.29	32	+	-	0.1059	lncRNA	RP11-1055B8.4
C3699340	chr3	27722433	27722781	96.52	71.27	61	.	-	0.0021	extended 5'-UTR	EOMES
C3381920	chr2	73986027	73986141	82.16	84.27	59	.	+	0.0132	lncRNA	AC073046.25
C5299720	chr8	144086406	144086461	77.68	76.67	16	.	+	0.0035	close to 3'-UTR	GPAA1
C1405001	chr1	38028048	38028083	85.08	61.73	49	-	+	0.0588	intergenic	
C3242841	chr22	50310214	50310266	70.02	67.48	14	-	-	0.0015	extended 3'-UTR	DENND6B
C5750650	chrX	111222341	111222439	84.96	62.60	58	-	+	0.0087	extended 3'UTR	PAK3
C3618380	chr3	173395924	173395957	72.44	75.08	46	+	+	0.0084	close to 5'-UTR	NLGN1
C3466931	chr3	114317904	114318189	100.32	84.26	82	-	-	0.0052	intergenic	
C1714780	chr1	54226057	54226170	82.21	69.45	59	+	-	0.0138	extended 3'-UTR	SSBP3
C0354200	chr11	133897157	133897283	94.81	66.05	58	.	-	0.0169	lncRNA	MIR4697HG
C5440070	chr8	88327680	88327769	75.01	69.45	56	+	-	0.0840	close to 5'-UTR	MMP16
C2046490	chr17	39563608	39563680	116.56	70.69	95	+	+	0.0588	intergenic	
C0778660	chr1	198888535	198888745	75.88	84.07	78	+	-	0.0145	lncRNA intronic	MIR181A1HG
C5220021	chr8	10887987	10888162	91.47	67.02	60	-	-	0.0190	intergenic	
C0992321	chr1	227919644	227919676	77.92	79.07	12	-	-	0.0111	extended 3'-UTR	WNT9A
C2196901	chr1	77068145	77068193	73.85	69.28	40	-	+	0.0272	lncRNA	RP4-564M11.2
C4897170	chr6	98843961	98844031	101.43	94.98	83	.	+	0.0840	intergenic	
C5927191	chrX	71172734	71172803	71.97	66.43	56	-	+	0.0084	close to 3'-UTR	NLGN3
C1764840	chr1	56995333	56995381	65.77	74.43	58	+	-	0.0116	extended 3'-UTR	DAB1
