# Supplementary material for: The identification and functional annotation of RNA structures conserved in vertebrates
Source: Genome Res. 2017 Aug;27(8):1371–83. doi: 10.1101/gr.208652.116 (PMC5538553; doi:10.1101/gr.208652.116)
Supplement: Supplemental Material [file supp_gr.208652.116_Supplemental_Table_S4.pdf]

Supplemental Table S4. Structure probing candidates. The four CRSs that have been structure probed in human and mouse. SI is the sequence identity between human and mouse sequence in the CRS alignment.

CRS region	CRS	chr	start	end	strand	pscore	FDR	RNA shape	SI [%]	location	gene name
C1785511	M1695693	chr15	82846711	82846804	-	77.75	11.03	[][]	45.45	close to 3'-UTR	HOMER2
C3699340	M0794543	chr3	27722453	27722502	+	73.74	21.69	[][]	51.14	extended 5'-UTR	EOMES
C0354200	M1486949	chr11	133897157	133897205	-	68.19	13.88	[][]	62.07	lncRNA	MIR4697HG
C2713000	M0698482	chr20	63403707	63403738	+	70.34	11.03	[]	56.25	extended 3'-UTR	KCNQ2
