# Supplementary material for: The identification and functional annotation of RNA structures conserved in vertebrates
Source: Genome Res. 2017 Aug;27(8):1371–83. doi: 10.1101/gr.208652.116 (PMC5538553; doi:10.1101/gr.208652.116)
Supplement: Supplemental Material [file supp_gr.208652.116_Supplemental_Table_S5.pdf]

Supplemental Table S5. CRS enrichment within gene regulatory regions. One-sided Z-test for enrichment of CMfinder predicted CRSs ("Predicted") and CRSs supported by unannotated transcript boundaries ("Transcribed") within gene regulatory regions. The statistical test considers only the search space for CRSs (human sequences covered by 17-species MULTIZ alignments). Compare to Figure 5A,E,I in the main manuscript.

Gene regulatory region	Fold enrichment	P-value
Predicted enhancer	1.13	$< 10^{-56}$
Transcribed enhancer	1.67	$< 10^{-185}$
Predicted 5' extensions	1.29	$< 10^{-94}$
Transcribed 5' extensions	1.83	$< 10^{-40}$
Predicted 3' extensions	1.06	$< 10^{-5}$
Transcribed 3' extensions	1.14	$< 10^{-4}$
Predicted mRNA 3' extensions	1.10	$< 10^{-7}$
Transcribed mRNA 3' extensions	1.15	0.0001
Predicted lncRNA 3' extensions	1.01	0.25
Transcribed lncRNA 3' extensions	1.08	0.10
