# Supplementary material for: The identification and functional annotation of RNA structures conserved in vertebrates
Source: Genome Res. 2017 Aug;27(8):1371–83. doi: 10.1101/gr.208652.116 (PMC5538553; doi:10.1101/gr.208652.116)
Supplement: Supplemental Material [file supp_gr.208652.116_Supplemental_Table_S6.pdf]

Supplemental Table S6. Significance of higher expression levels of structured versus unstructured regulatory regions. Listed are number of tissues with significantly higher expression level of structured loci (overlapped by CRSs) than unstructured loci ($P < 0.05$, Mann-Whitney U-test BH corrected) for bins of different GC content / phastCons score. The considered enhancers and TSS upstream regions had to be supported by DNaseI hypersensitive sites and bi- or unidirectional FANTOM5 CAGE expression as described Methods and Supplemental Fig. 10. Alternative poly(A) sites had to be supported by experimental polyadenylation signals. Total RNA-seq is from the ENCODE project, comprises 19 tissues and 2 biological replicates for each tissue which both had to be significant to be counted. Poly(A)-selected RNA-seq is from Illumina Body Map 2.0, comprises 16 tissues and no replicates.

Nr. tissues	GC content	phastCons score	structured loci	unstructured loci
total RNA-seq for enhancers				
19	(0,1]	(0,1]	2862	7248
8	(0.25,0.5]	(0.,0.08]	625	3178
6	(0.25,0.5]	(0.16,0.24]	145	343
19	(0.5,0.75]	(0.,0.08]	704	1935
18	(0.5,0.75]	(0.08,0.16]	325	300
18	(0.5,0.75]	(0.16,0.24]	176	118
6	(0.5,0.75]	(0.24,1]	292	99
total RNA-seq for TSS upstream regions				
18	(0,1]	(0,1]	354	912
2	(0.25,0.5]	(0.24,1]	12	17
7	(0.5,0.75]	(0.,0.08]	124	368
2	(0.5,0.75]	(0.24,1]	39	12
poly(A)-selected RNA-seq for alternative poly(A) sites				
16	(0,1]	(0,1]	740	3366
3	(0.,0.25]	(0.,0.08]	3	29
3	(0.,0.25]	(0.16,0.24]	1	6
16	(0.25,0.5]	(0.,0.08]	299	1978
16	(0.25,0.5]	(0.08,0.16]	121	551
9	(0.25,0.5]	(0.16,0.24]	59	159
16	(0.25,0.5]	(0.24,1]	90	216
14	(0.5,0.75]	(0.,0.08]	101	326
8	(0.5,0.75]	(0.08,0.16]	32	44
3	(0.5,0.75]	(0.16,0.24]	9	17
