# Supplementary material for: The identification and functional annotation of RNA structures conserved in vertebrates
Source: Genome Res. 2017 Aug;27(8):1371–83. doi: 10.1101/gr.208652.116 (PMC5538553; doi:10.1101/gr.208652.116)
Supplement: Supplemental Material [file supp_gr.208652.116_Supplemental_Table_S7.pdf]

Supplemental Table S7 List of public CLIP data. The listed CLIP-seq libraries have been used in the study to find CRSs potentially targeted by RNA binding proteins (RBPs) in human and mouse.

	Human		Mouse	
RBP	PubmedID	NCBI Accession	PubmedID	NCBI Accession
AGO1	20371350	GSM545212		
AGO2	20371350	GSM545213		
AGO3	20371350	GSM545214		
AGO4	20371350	GSM545215		
ALKBH5	22681889	GSM936506		
NCBP3	22681889	GSM936507		
RTCB	22681889	GSM936508		
CAPRIN1	22681889	GSM936509		
CTCF	24696455	GSM1296147		
DKC1	23706177	GSM1067866		
DGCR8	22796965	GSM955512, GSM955510		
EIF4A3	23085716	GSM1001330, GSM1001331		
EIF3A	25849773	GSM1585664, GSM1585665, GSM15856656		
EIF3B	25849773	GSM1585667, GSM1585668, GSM1585669		
EIF3D	25849773	GSM1585670, GSM158567, GSM158562		
EIF3G	25849773	GSM1585673, GSM1585674, GSM15856735		
ELAVL1	21723170	GSM738185		
EWSR1	22081015			
FBL	23706177	GSM1067864		
FMR1	22213601	GSM977615	21784246	GSM1098051
RBFOX2	19136955		24837674	SRP041098
FUS	22081015		22934129	
FXR1	22213601	GSM977619		
FXR2	22213601	GSM977620		
HNRNPA1	22574288	GSM859978, GSM859979, GSM85980, GSM85981		
HNRNPA2B1	22574288	GSM859982		
HNRNPC	20601959	GSM630861		
HNRNPD	25366541	GSM1279664, GSM1279665, GSM1279666, GSM127967		
HNRNPF	22574288	GSM859983, GSM859984, GSM859985, GSM859986		
HNRNPH	21057496	GSM581052, GSM581051		
HNRNPL	24526010	GSM921620, GSM921621, GSM921622		
HNRNPM	22574288	GSM859987, GSM859988		
HNRNPU	22574288	GSM859989, GSM859990, GSM859991, GSM859992		
IGF2BP1	20371350	GSM545206		
IGF2BP2	20371350	GSM545208	27385015	GSE83822
IGF2BP3	20371350	GSM545209		
LARP4B	26001795	GSM1694273		
LIN28A	23481595	GSM1087848	23102813	GSM910957
LIN28B	23481595	GSM108789, GSM10878450, GSM108784851		
METTL3	24407421	GSM1223896		
MOV10	24726324	GSM1173263		
MSI1	26100017	GSM1681975, GSM1681976, GSM16819757		
NOP56	23706177	GSM1067863		
NOP58	23706177	GSM1067862, GSM1067861		
NSUN2	23871666	GSE44385		
PRKRA	25416952	GSM1548748, GSM1548749, GSM1548750		
PTBP1	20064465	GSM480476, GSM480477		
PUM2	20371350	GSM545210		
QKI	20371350	GSM545211		
RBM4	22678294	GSM885044		
RBM10	24000153	GSM1095143, GSM1095142		
RBM47	24898756	GSM1409655, GSM1409656		
RBPMS2	24860013			
RBPMS	24860013			
SRSF1	19116412		23562324	GSM1088390
TAF15	22081015			
TARBP2	25416952	GSM1548746, GSM1548747		
TARDBP	21358640		21358643	GSM672062
TIA1	21048981			
TIAL1	21048981			
TNRC6A	20371350	GSM545218		
TNRC6B	20371350	GSM545219		
TNRC6C	20371350	GSM545220		
U2AF65	23152763		22934129	
UPF1	24726324	GSM1173266	23766421	GSM1024301
WTAP	24407421	GSM1223897		
ZC3H7B	22681889	GSM936510		
