# Supplementary material for: The identification and functional annotation of RNA structures conserved in vertebrates
Source: Genome Res. 2017 Aug;27(8):1371–83. doi: 10.1101/gr.208652.116 (PMC5538553; doi:10.1101/gr.208652.116)
Supplement: Supplemental Material [file supp_gr.208652.116_Supplemental_Table_S8.pdf]

**Supplemental Table S8.** List of public RNA-seq libraries. The listed RNA-seq libraries have been used in the study to assign expression levels to CRS regions and gene regulatory regions. For the first two datasets hg38 and mm10 premapped BAM files were downloaded from the indicated URLs: ENCODE phase 3 was mapped with STAR 2.5.1b; Illumina Human Body Map 2.0 (E-MTAB-513) was mapped with BWA v0.6.2-r126-tpx. For the third dataset (SRX081) we downloaded raw reads (.fastq files) and their processing is described in the Methods. Using the ENCODE phase 3 total RNA for human-mouse comparison we matched the RNA-seq data from mouse forebrain with human diencephalon, and mouse hindbrain with human cerebellum.

| ENCODE phase 3: rRNA-depleted Total RNA (>200bp), stranded PE101 Illumina HiSeq 2000<br>URL: https://www.encodeproject.org/files/ | | | |
|---|---|---|---|
| Species | Tissue | Library name | Replicate |
| Human | cameratypeeye | ENCFF841MML | 1 |
| | | ENCFF604QYU | 2 |
| | cerebellum | ENCFF883NIZ | 1 |
| | | ENCFF730CJE | 2 |
| | diencephalon | ENCFF164JEV | 1 |
| | | ENCFF811MSS | 2 |
| | frontalcortex | ENCFF029BEL | 1 |
| | | ENCFF542GSD | 2 |
| | heart | ENCFF575PML | 1 |
| | | ENCFF413CTV | 2 |
| | liver | ENCFF741AOJ | 1 |
| | | ENCFF444EKY | 2 |
| | lung | ENCFF816ZAP | 1 |
| | | ENCFF202ETQ | 2 |
| | metanephros | ENCFF701CAC | 1 |
| | | ENCFF841GMR | 2 |
| | occipital | ENCFF491VZY | 1 |
| | | ENCFF992HMB | 2 |
| | parietallobe | ENCFF376UOL | 1 |
| | | ENCFF090FOY | 2 |
| | skeletalmuscle | ENCFF997MIE | 1 |
| | | ENCFF422EVZ | 2 |
| | skinofbody | ENCFF017ZWH | 1 |
| | | ENCFF969GXB | 2 |
| | spinalcord | ENCFF976QSN | 1 |
| | | ENCFF595LQV | 2 |
| | stomach | ENCFF559BDS | 1 |
| | | ENCFF995BPY | 2 |
| | temporallobe | ENCFF196YLT | 1 |
| | | ENCFF460PJA | 2 |
| | thyroidgland | ENCFF756NWQ | 1 |
| | | ENCFF274JNT | 2 |
| | tongue | ENCFF019KCS | 1 |
| | | ENCFF549UBP | 2 |
| | urinarybladder | ENCFF514QIL | 1 |
| | | ENCFF719GEV | 2 |
| | uterus | ENCFF535JQR | 1 |
| | | ENCFF979LYG | 2 |
| Mouse | heart | ENCFF054EUK | 1 |
| | | ENCFF228QVL | 2 |
| | liver | ENCFF162OCN | 1 |
| | | ENCFF443YYY | 2 |
| | forebrain | ENCFF965MHF | 1 |
| | | ENCFF203VYY | 2 |
| | hindbrain | ENCFF772VMM | 1 |
| | | ENCFF172HHP | 2 |

E-MTAB-513 (Asmann et al.): Illumina Human Body Map 2.0, polyA-selected (50bp PE unstranded)
URL: ftp://ftp.ensembl.org/pub/data_files/homo_sapiens/GRCh38/rnaseq/

| Species | Tissue | Replicate |
|---|---|---|
| Human | adipose | 1 |
| | adrenal | 1 |
| | blood | 1 |
| | brain | 1 |
| | breast | 1 |
| | colon | 1 |
| | heart | 1 |
| | kidney | 1 |
| | liver | 1 |
| | lung | 1 |
| | lymph | 1 |
| | ovary | 1 |
| | prostate | 1 |
| | skeletal muscle | 1 |
| | testes | 1 |
| | thyroid | 1 |

| SRX081 (Brawand et al.): polyA-selected (250-300bp unstranded), Illumina Genome Analyzer IIx<br>URL: ftp://ftp-trace.ncbi.nlm.nih.gov/sra/sra-instant/reads/ByExp/sra/SRX/SRX081/ | | | |
|---|---|---|---|
| Species | Tissue | Library name | Replicate |
| Human | testis | SRR306858 | 1 |
| | | SRR306857 | 2 |
| | liver | SRR306856 | 1 |
| | | SRR306854 | 2 |
| | | SRR306855 | 3 |
| | kidney | SRR306853 | 1 |
| | | SRR306852 | 2 |
| | | SRR306851 | 3 |
| | heart | SRR306850 | 1 |
| | | SRR306848 | 2 |
| | | SRR306849 | 3 |
| | | SRR306847 | 4 |
| | cerebellum | SRR306845 | 1 |
| | | SRR306846 | 2 |
| | | SRR306844 | 3 |
| | brain | SRR306843 | 1 |
| | | SRR306842 | 2 |
| | | SRR306841 | 3 |
| | | SRR306840 | 4 |
| | | SRR306839 | 5 |
| | | SRR306838 | 6 |
| Mouse | testis | SRR306776 | 1 |
| | | SRR306775 | 2 |
| | liver | SRR306774 | 1 |
| | | SRR306773 | 2 |
| | | SRR306772 | 3 |
| | kidney | SRR306771 | 1 |
| | | SRR306770 | 2 |
| | | SRR306769 | 3 |
| | heart | SRR306768 | 1 |
| | | SRR306767 | 2 |
| | | SRR306766 | 3 |
| | cerebellum | SRR306765 | 1 |
| | | SRR306764 | 2 |
| | | SRR306763 | 3 |
| | brain | SRR306762 | 1 |
| | | SRR306758 | 2 |
| | | SRR306759 | 3 |
| | | SRR306760 | 4 |
| | | SRR306761 | 5 |
| | | SRR306757 | 6 |
