# Supplementary material for: The identification and functional annotation of RNA structures conserved in vertebrates
Source: Genome Res. 2017 Aug;27(8):1371–83. doi: 10.1101/gr.208652.116 (PMC5538553; doi:10.1101/gr.208652.116)
Supplement: Supplemental Material [file supp_gr.208652.116_Supplemental_Table_S9.pdf]

Supplemental Table S9. List of primers for qRT-PCR. List of primers used for validating CMfinder predicted CRS regions in seven tissues in human and mouse by qRT-PCR.

CRS region	Forward Primer	Reverse Primer
	Human	
C0778660	GACTGCATGGCTACAGAGGGCTATAA	ACATGGTGTTCACAGAATGGCTTAGATTA
C0927120	ATATGTCACCTTTCCTGGGATTACTTTC	TATGTGGTCCATGGTGTGTTGAA
C0992321	TGCCAGCATGCATTTATACAACACACGCT	GGAGGCAGTCTTGGGCTCGCATCT
C1405001	AATATATCAAGGGAACTGCTGTGTTTAGG	ACTGGGAGAAAGCTGTAAATGACTCA
C1714780	GCAGGATTCTCACTGGGAAGACTGGG	TTTTGTTTGGGGTTTTACCATTGTTGGGA
C1764840	TGCGGCATGTAAAGATTGAAGAGCTATGG	GGGGAGTCTGCACGGTTCTGGTTAGAT
C1785511	TCTAATGTGATGGCAGGCAGGAGAGATGA	CTAGTGCCGCCCGGGGAATGTAAA
C2046490	ATCAGCGCCGAATTGCCATCTCCTTGAGC	CTCTCCCTTCCTCCTCGGCGTTTGCACA
C2196901	TCACCTCTACCAGAATGTGAAGGGAAAGG	TTGCCCTCTCCTGTAGCTGGGATGTA
C2213410	CAGCGACACGTGTAAACACCGCCAAACAG	AACTGCCCTGCTTGGGGACAATGCTCA
C2713000	GTGCACTCTAACAAACACTGCGGGAGC	CTCTAGCCTGAGCAGTGCAGCCACCTACT
C3242841	GGCCTCTATGTGTTCTGTTTCATGTGGTC	GTACATGTCAACTTGTCCAGCCCAACTG
C3381920	CCCCACCACACCCCTACCTGCTCAACT	CCCTCTTCACCTCCTCTGCAGCAGTCTGA
C3466931	CCAGAAAGGAATTTGTTTGTCTTGGT	CCTAAGGCTGTTCAATAGCTTTTCCATAT
C3618380	GATTCAGACTCCTGCACCCGACAA	GTATCCAGAGATTTCGTACCAGGAGCCAC
C3699340	AAGTGCGCGCCAGGCAGGTTCACTG	CCTCCTGCTTTCCAACTCACGGCGCTTTC
C4897170	CTATTTACTCGCCTCTGCCTGTAGATTTC	TTAATGTATACTGGTAATCAGCGATGCGT
C5220021	CACTACCTCAGCTGCCTAAGGAAACTCAC	AGGGAAGAAAGGACTGAGAGAAAGGGAA
C5299720	GGGCTGTGTGCATTGCTCTGTGGTACTGT	GGGCATCTCACCTGGGCTGACTCAACT
C5440070	GGCGAAGACAGGGTCAGCAGTAGTT	CGGGAAGGAGGAGTTATGTAGATTACGG
C5750650	GCCAAGGTACATCCATCCATCTAAC	TTTTCTTTTCACATTTGTTGTTTTATCCC
C5927191	GCTGATGAGGTAACATTCAATGGTTG	ACTTGATCTCATTTTCCCCTCTATCATTC
	Mouse	
C0354200	GTGTGCATGAGCTCACCTGT	GGGAAAAGCACCTGTTTGAG
C0778660	AAGAGACACAAGGCCTAGCACCAA	AAGCCCTGCTCCATAAAGTGTGTG
C0927120	TCTTTCTCCCTGCTCTGAGG	AGCTGTCCATCTGCTGTTGA
C0992321	GTGTATATTCCGAGCTGGCATGGT	TAGTGCCTGTTGTCATCTCTGCCA
C1405001	AAGTTGTCCTGTCACTGCCTCCTT	AGATGGAGACTAGGCCAGCAGT
C1714780	TATGGAGGTCAGGGCTATGG	TCCAGCGTACACACTGTTCC
C1764840	AAGAGGAGGAGGAAGCAAGC	TTGGCAGAATACTGGTTTTGG
C1785511	AGTGACAGCTCTTGGGGAGA	AACCTCAGAGCCCTTTAGCC
C2046490	TTGCCATCTCCTTGAGCTCTCTGT	CTCCTCAGCATTTGCACACACCAA
C2196901	GCCCAAGCTCAGGAAGATACCATT	TGAAGGACAGAGCAAGAAGGGAAC
C2213410	TAGCTGTACTTCCCGCAAGG	GACCCTGCTTTGGAACAAGT
C2713000	CCTGTTGTATGTATTCTTGTGTGTGC	ATGTGACCAAGTATGGCTGAAGGC
C3242841	CACACTGCCCACTCTACAGG	GGTGCCATGGTTCTGAAGAT
C3381920	AGATTTCATCTTGGCTGGGTGGGT	CACCCAGTGATGCACTTCCTCTTT
C3466931	GAAGGGAAAAGGGAAACACC	GGAGGCCTCTCTTCAGCTTT
C3618380	TGTGCTACAAGGCTGTCAGG	GCACACACACATCAGCCTCT
C3699340	AATGGCTGGTTGCTAAATGG	CCTTCCGCAAGAAAACAAAC
C4897170	GCTTGAATGCTTAGCGCAAT	GGGCAGGGTGGTACACAA
C5220021	ATTGTCCTGTGACCCCAGTC	GCAGCTGGAAGTTCAGAAGG
C5299720	CCCAGCTCCATCAACTTCTG	CCCAGAATCGCTTCTAGCAC
C5440070	ACTGCTCCTTCCCTCCTCTC	TGTGTTTCGGGTGAGTGAGT
C5750650	AATCACCCTGTTCTCTGCCTCCTT	CTAGACCTGGCTGTTGTCACCTTA
C5927191	GCAGCTACTGGTGGGAGTTT	TCTGTGTGCTGCAATCCTTC
