# Supplementary material for: The identification and functional annotation of RNA structures conserved in vertebrates
Source: Genome Res. 2017 Aug;27(8):1371–83. doi: 10.1101/gr.208652.116 (PMC5538553; doi:10.1101/gr.208652.116)
Supplement: Supplemental Material [file supp_gr.208652.116_Supplemental_Table_S10.pdf]

**Supplemental Table S10.** List of primers and house-keeping genes for qRT-PCR. House-keeping genes and their primers sequences used for normalization in qRT-PCR.

| Gene | Forward Primer | Reverse Primer |
| --- | --- | --- |
| | Human house-keeping genes | |
| ALAS-1 | CCGTGAGGAAAGAGGTTGCT | GTCATTACTGCACCAGACTGACACT |
| B2M | TGTGCTCGCGCTACTCTCTC | CTGAATGCTCCACTTTTTCAATTCT |
| G6PD | CCGGGACAACATCGCCTGCGTTATC | ACGGCTGCAAAAGTGGCGGTGGT |
| GAPDH | GGAAGGTGAAGGTCGGAGTCAA | GATCTCGCTCCTGGAAGATGGT |
| EiF6 | AAGTCTTCAGACAGACAGTGGCCGACCAG | ACCACCATCCCAGCAGCAATCACCT |
| EEF1D1A | AGAGCCAGAGAGAACATCCAGAAATCCCT | CTCCTCCTCATTGTCACTGCCAAACAG |
| | Mouse house-keeping genes | |
| Actb | ATGGAGGGGAATACAGCCC | TTCTTTGCAGCTCCTTCGTT |
| Gapdh | CGTCCCGTAGACAAAATGGT | TTGATGGCAACAATCTCCAC |
| Ppia | CACCGTGTTCTTCGACATCA | CAGTGCTCAGAGCTCGAAAGT |
