# Supplementary material for: The identification and functional annotation of RNA structures conserved in vertebrates
Source: Genome Res. 2017 Aug;27(8):1371–83. doi: 10.1101/gr.208652.116 (PMC5538553; doi:10.1101/gr.208652.116)
Supplement: Supplemental Material [file supp_gr.208652.116_Supplemental_Table_S11.pdf]

Supplemental Table S11. List of primers for structure probing.

CRS	Species	Forward Primer	Reverse Primer
M0112740	hg18	TAATACGACTCACTATAGGCCGGAACTCCAGCCCCCCAAG	CCAGCCCCTCCTCTGCCC
	mm8	TAATACGACTCACTATAGGCCCTGCCAGTGCGCACTATG	CACTGGGGCCTCCAGTCTTTC
M0458670	hg18	TAATACGACTCACTATAGGCCAGCCGGCATCAGCGCC	GATTTCTCTTAATCTTCCTCCGCC
	mm8	TAATACGACTCACTATAGGCGGTATCAGCGCCGAATTGC	CTCCCTTCCTCCTCAGCATTTG
M1164019	hg18	TAATACGACTCACTATAGGCCTTCCCTCCTCTCCTCGC	CGCGTGCGTGTGTGTCGG
	mm8	TAATACGACTCACTATAGGCTCCTTCCCTCCTCTCTGCTC	GTGTGTTTCGGGTGAGTGAGTATG
M0698482	hg18	TAATACGACTCACTATAGGTATACAGGTGTGGTCAGTGC	CACACACACGTGCTCACATAC
	mm8	TAATACGACTCACTATAGGTCTTGTGTGTGCATATGAAAATG	GCTGAAGGCCAATGGCTGC
M0203048	hg18	TAATACGACTCACTATAGGCTGATTGAGAGTGTAGCTG	TGGCTCTGGGAGTGGG
	mm8	TAATACGACTCACTATAGGGGCCCTTCACAGAGAGATAC	ACCCAGGTTGGCCTGG
M1486949	hg18	TAATACGACTCACTATAGGGTGGGAATGAAGGGGGGATG	GTGGCTGGGGGTGCCTG
	mm8	TAATACGACTCACTATAGGCTGTGGGAGTGAGGGG	CCATGGACAGGCCCTTC
M0794543	hg18	TAATACGACTCACTATAGGCCCGCGCCGGGGCTAC	CGAGGAAGGAAAAGGAGAGG
	mm8	TAATACGACTCACTATAGGGAGGGGAAGGTAATGTC	GCGGAAGGAAAGGGGC
M0056174	hg18	TAATACGACTCACTATAGGCTCACCAATCTCTTTCTTC	CCGCAGAGGTTAAAGGGAC
	mm8	TAATACGACTCACTATAGGTCTCCAACCTCTCTCTTCCCTC	CCCAGGCAGTGTGTGTGTGC
M0794543	hg18	TAATACGACTCACTATAGGCCCGCGCCGGGGCTAC	CGAGGAAGGAAAAGGAGAGGGC
	mm8	TAATACGACTCACTATAGGGAGGGGAAGGTAATGTCCCC	GCGGAAGGAAAGGGGC
M2089837	hg18	TAATACGACTCACTATAGGGAGGTACGAGGAAGGAAAAGG	CCCGCGCCGGGGCTACCC
	mm8	TAATACGACTCACTATAGGCCGCGGCCTGGTTATAAAGG	GGTTTGTTTTCTTGCGGAAGGAAAG
